# Liver‐directed gene therapy for ornithine aminotransferase deficiency

**DOI:** 10.15252/emmm.202217033

**Published:** 2023-01-17

**Authors:** Iolanda Boffa, Elena Polishchuk, Lucia De Stefano, Fabio Dell'Aquila, Edoardo Nusco, Elena Marrocco, Matteo Audano, Silvia Pedretti, Marianna Caterino, Ilaria Bellezza, Margherita Ruoppolo, Nico Mitro, Barbara Cellini, Alberto Auricchio, Nicola Brunetti‐Pierri

**Affiliations:** ^1^ Telethon Institute of Genetics and Medicine (TIGEM) Pozzuoli Italy; ^2^ Department of Pharmacology and Biomolecular Sciences University of Milan Milan Italy; ^3^ Department of Molecular Medicine and Medical Biotechnology University of Naples “Federico II” Naples Italy; ^4^ CEINGE – Biotecnologie Avanzate s.c.a.r.l. Naples Italy; ^5^ Department of Experimental Medicine, Section of Physiology and Biochemistry University of Perugia Perugia Italy; ^6^ Department of Experimental Oncology, IEO European Institute of Oncology IRCCS Milan Italy; ^7^ Department of Advanced Biomedical Sciences “Federico II” University Naples Italy; ^8^ Department of Translational Medicine “Federico II” University Naples Italy; ^9^ Scuola Superiore Meridionale (SSM, School of Advanced Studies), Genomics and Experimental Medicine Program University of Naples Federico II Naples Italy

**Keywords:** AAV, gyrate atrophy of choroid and retina, inherited metabolic diseases, liver gene therapy, ornithine aminotransferase, Genetics, Gene Therapy & Genetic Disease

## Abstract

Gyrate atrophy of choroid and retina (GACR) is a chorioretinal degeneration caused by pathogenic variants in the gene encoding ornithine aminotransferase (OAT), an enzyme mainly expressed in liver. Affected patients have increased ornithine concentrations in blood and other body fluids and develop progressive constriction of vision fields leading to blindness. Current therapies are unsatisfactory and better treatments are highly needed. In two mouse models of OAT deficiency that recapitulates biochemical and retinal changes of GACR, we investigated the efficacy of an intravenously injected serotype 8 adeno‐associated (AAV8) vector expressing OAT under the control of a hepatocyte‐specific promoter. Following injections, OAT‐deficient mice showed reductions of ornithine concentrations in blood and eye cups compared with control mice injected with a vector expressing green fluorescent protein. AAV‐injected mice showed improved electroretinogram response and partial restoration of retinal structure up to one‐year post‐injection. In summary, hepatic OAT expression by AAV8 vector was effective at correction of hyperornithinemia and improved function and structure of the retina. In conclusion, this study provides proof‐of‐concept of efficacy of liver‐directed AAV‐mediated gene therapy of GACR.

## Graphical abstract

Deficiency of ornithine aminotransferase (OAT) in liver results in increased ornithine concentrations and retinal toxicity, especially the retinal pigment epithelium (RPE). This study investigated liver‐directed gene transfer for OAT deficiency.

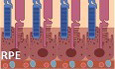

## Introduction

Ornithine‐δ‐aminotransferase (OAT) deficiency results in gyrate atrophy of the choroid and retina (GACR), a rare autosomal recessive inborn error of metabolism with progressive retinal degeneration. OAT is a pyridoxal‐phosphate‐dependent mitochondrial enzyme expressed mostly in liver that converts ornithine into pyrroline‐5‐carboxylic acid (P5C) (Brody *et al*, 1992). In newborns or infants, the enzyme reaction catalyzed by OAT is toward the synthesis of ornithine, whereas later in life the enzyme catalyzes the opposite reaction of ornithine conversion into P5C. Therefore, deficiency of OAT activity in newborns can cause hyperammonemia because of deficiency in synthesis of ornithine, that is required by the urea cycle (Cleary *et al*, 2005). In contrast, in adults OAT deficiency results in increased ornithine and reduced synthesis of P5C. Increased ornithine concentrations are thought to be responsible of progressive retinal degeneration by a yet unknown mechanism (Wang *et al*, 2000). Although most patients with OAT deficiency are diagnosed in early adolescence, age of onset and severity of GACR are variable. The most common manifestations of GACR include night blindness and visual field restriction evolving into extensive retinal atrophy with reduction in central vision. The long‐term prognosis of GACR is poor with complete blindness in most cases by the age of 40 years (Takki & Milton, 1981; Kaiser‐Kupfer *et al*, 1985). The diagnosis is based on increased blood ornithine concentrations, that are typically 10‐to‐20 fold higher than normal. GACR can be potentially diagnosed pre‐symptomatically by newborn screening (de Sain‐van der Velden *et al*, 2012) that might allow early and more effective interventions before the onset of retinal damage. Only a small proportion of patients are responsive to treatment with pyridoxine (vitamin B6), the cofactor of OAT (Michaud *et al*, 1995). A diet restricted in arginine, the precursor of ornithine started at an early age and resulting in long‐term reduction of plasma ornithine concentrations appears to slow down the progression of chorioretinal lesions and the loss of retinal function (Kaiser‐Kupfer *et al*, 2002). However, the outcomes are inconsistent (Vannas‐Sulonen *et al*, 1987) and the diet suffers of poor long‐term compliance.

As liver‐directed gene therapy by AAV vectors is showing safety and efficacy in a growing number of clinical trials, several inborn errors of metabolism are being considered as potential disease targets. Given the cumbersome dietary treatment as the only currently available therapy, GACR could be particularly attractive as disease target if correction of the hyperornithinemia can be effective in prevention of the retinal degeneration. OAT deficiency does not result in hepatic injury and there are no known GACR patients who underwent liver transplantation. Therefore, we investigated whether restoration of OAT activity by gene trasfer to the liver can correct the retinal disease. Given that the liver of GACR patients is structurally normal, there is potential for efficient AAV‐mediated liver gene transfer and sustained OAT expression. In addition, the coding sequence of the gene encoding OAT is 1,320 bp in size (Sergouniotis *et al*, 2012) and thus, it can be accommodated into AAV vectors. In the present study, we provide a proof‐of‐concept for the efficacy of liver‐directed gene therapy for GACR that might pave the way toward a clinical trial.

## Results

**Figure EV1:**
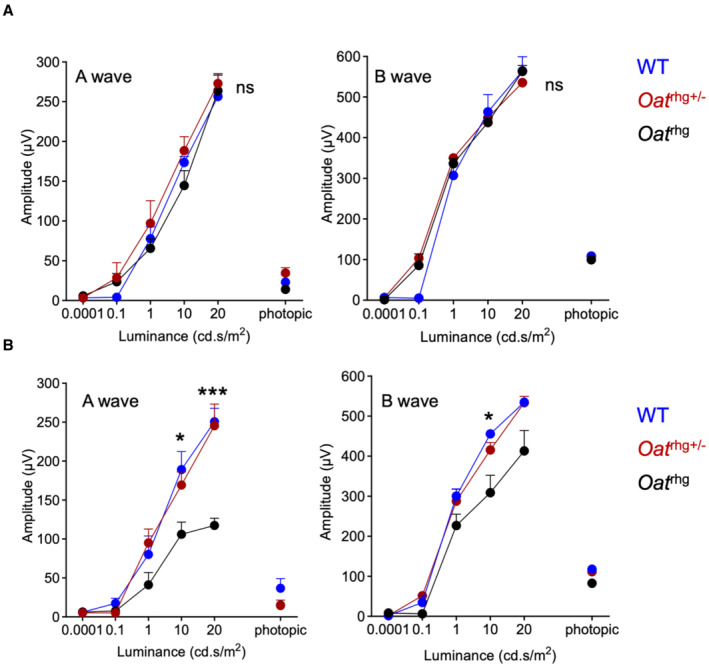
Retinal function in *Oat* ^*rhg*^ mice A, Ba‐ and b‐wave amplitudes under scotopic and photopic conditions in *Oat* ^*rhg*^ pigmented mice. The amplitudes induced by increasing light intensities under scotopic conditions in 7‐month‐old (A) and 12‐month‐old (B) wild‐type (blue circles, *n* = 6), heterozygous *Oat* ^*+/rhg*^ (red circles, *n* = 6) and homozygous *Oat* ^*rhg*^ mice (black circles, *n* = 6). Data are shown as means ± SEM. Two‐way ANOVA, *P < 0.05; ***P < 0.001. *Abbreviation: ns: not statistically significant difference*.

B6Ei; AKR‐rhg (*Oat* ^*rhg*^) mice carry a homozygous missense variant in the *Oat* gene, changing a glycine into alanine at the amino acid position 353, that results in deficiency of OAT activity. The *Oat* ^*rhg*^ mice are in a mixed pigmented (C57BL/6JEi) and albino (AKR) background, have increased blood ornithine concentrations (Bisaillon *et al*, 2014) and develop retinal degeneration by 12 months of age as shown by electroretinogram (ERG), whereas no abnormalities were detected at 7 months of age (Fig EV1A and B).

**Figure EV2:**
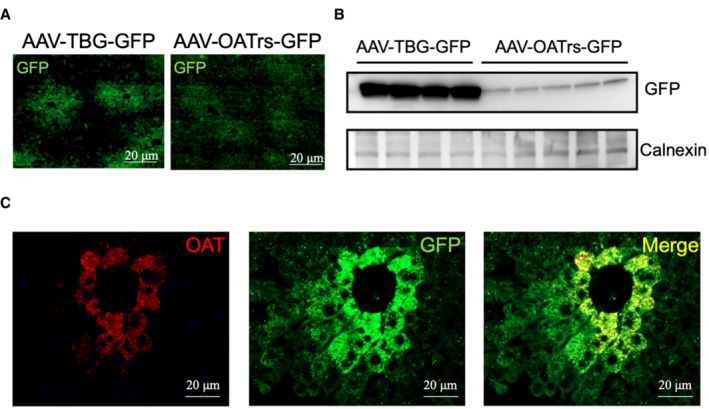
Patterns of liver transduction Liver section from wild‐type C57BL/6N mice systemically injected with an AAV8 vector driving GFP expression under the control of the TBG promoter (AAV‐TBG‐GFP) or under the control of the OAT regulatory sequence (AAV‐OATrs‐GFP). Representative images from experimental groups including at least *n* = 4 mice per group are shown. Scale bar: 20 μm.Western Blot analysis of lysates from livers of wild‐type C57BL/6N mice injected with AAV‐TBG‐GFP (*n* = 4) or AAV‐OATrs‐GFP (*n* = 5) vectors.Representative immunofluorescence images of livers from wild‐type C57BL/6N mice (*n* = 4) injected with AAV‐TBG‐GFP. OAT staining is show in red and GFP in green, colocalization of the signal is shown in yellow. Scale bar: 20 μm.

Liver expression of OAT is highly zonated with a pericentral pattern (Halpern *et al*, 2017). Therefore, we first evaluated the pattern of expression of both the thyroxine‐binding globulin (TBG) and OAT promoters driving green fluorescent protein (GFP) expression delivered by a serotype 8 AAV vector in wild‐type C57BL/6N mice. Mice were injected intravenously (i.v.) with AAV vectors expressing GFP under the control of either the TBG (*n* = 4) or the OAT regulatory sequence (Engelhardt *et al*, 1991; *n* = 5) at the dose of 1 × 10^13^ genome copies (gc)/kg and hepatic GFP expression was evaluated at 4 weeks post‐injection. Greater expression of GFP was detected in livers of mice injected with AAV‐TBG‐GFP vector by both immunofluorescence and western blot analyses (Fig EV2A and B). To ensure TBG promoter was effective in driving OAT expression in hepatocytes that physiologically express OAT (i.e., pericentral hepatocytes), we evaluated by immunofluorescence co‐localization of GFP and OAT in livers of wild‐type C57BL/6N mice injected with AAV‐TBG‐GFP. OAT and GFP proteins showed indeed an overlapping expression in pericentral hepatocytes (Fig EV2C).

**Figure EV3:**
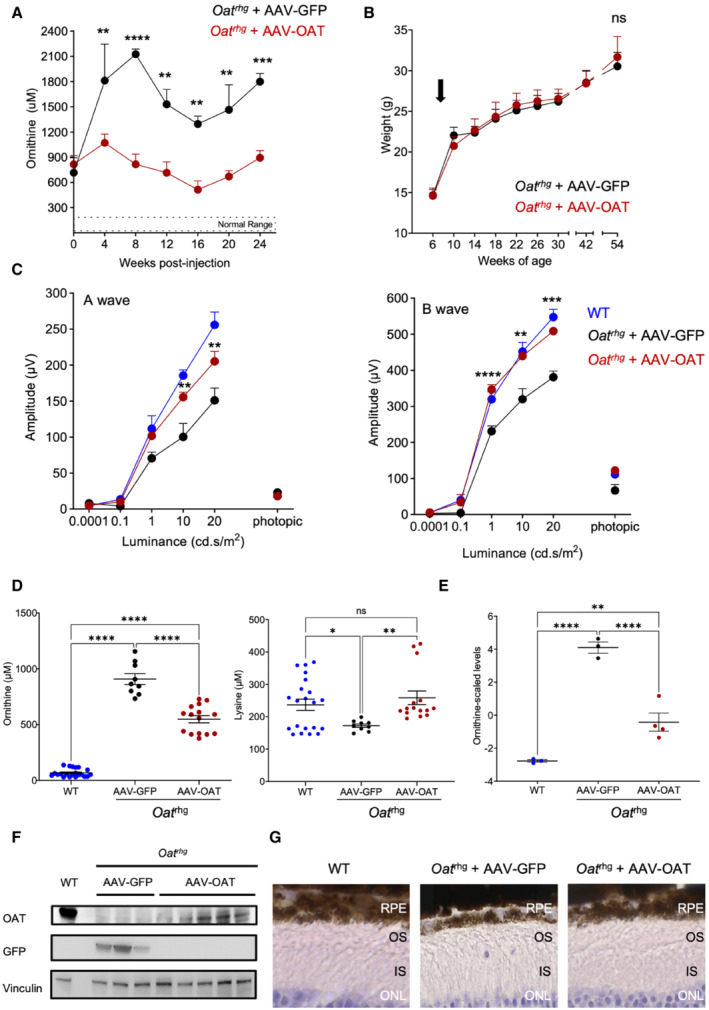
OAT gene transfer in *Oat* ^*rhg*^ pigmented mice injected at 6 weeks of age improves retinal phenotype Plasma ornithine concentrations in *Oat* ^*rhg*^ mice injected with AAV‐OAT (red circles, *n* = 5) or AAV‐GFP (black circles, *n* = 3) vectors. Averages ± SEM are shown; the two‐way ANOVA test was used to perform a statistical comparison between groups; **P < 0.01; ***P < 0.001, ****P < 0.0001.Body weights of *Oat* ^*rhg*^ mice injected at 6 weeks of age; the arrow indicates the time of the injections with AAV‐OAT (red circles, *n* = 5) or AAV‐GFP (black circles, *n* = 3) vectors; data are shown as means ± SEM; Two‐way ANOVA test was used for groups comparison.a‐ and b‐waves amplitudes recorded in scotopic conditions plotted as a function of light intensity (log cd * s/m^2^) in eyes of 11‐month‐old *Oat* ^*rhg*^ mice injected with AAV‐OAT (*n* = 9, red circles) or AAV‐GFP (*n* = 4, black circles) vectors. Wild‐type (WT) mice were used as controls (*n* = 10, blue circles). All data are shown as averages ± SEM. Two‐way ANOVA test was used for comparison between groups. **P < 0.01; ***P < 0.001, ****P < 0.0001.Plasma ornithine and lysine concentrations of *Oat* ^*rhg*^ mice at 12 months post‐injection measured by HPLC. One‐way ANOVA test with Tukey correction was used for comparison between groups; data are shown as averages ± SEM. *P < 0.05; **P < 0.01; ****P < 0.0001.Ornithine concentrations in the eyes of mice injected with AAV‐OAT compared with eyes of either wild‐type or AAV‐GFP controls. Data are shown as z‐scores. Means ± SEM are shown. One‐way ANOVA with False Discovery Rate correction was used for comparison between groups. **P < 0.01, ****P < 0.0001.Western blotting for OAT and GFP on livers of *Oat* ^*rhg*^ mice harvested 12 months after the injections of AAV‐OAT or AAV‐GFP vectors. A WT mouse was included as control, and Vinculin was used as loading control.Morphological analysis of *Oat* ^*rhg*^ mice retinas 12 months post‐injection of AAV‐GFP or AAV‐OAT. An age‐matched WT mouse is shown as control (left panel). Semi‐thin sections (40×). Abbreviations: ns, not statistically significant difference; RPE, retinal pigment epithelium; OS, outer segment; IS, inner segment; ONL, outer nuclear layer; WT, wild‐type.

Next, we injected i.v. 6‐week‐old *Oat* ^*rhg*^ pigmented mice with an AAV vector expressing the human OAT under the control of the TBG promoter (AAV‐OAT) at the dose of 1 × 10^13^ gc/kg (*n* = 5), whereas age‐matched control *Oat* ^*rhg*^ mice (*n* = 3) were injected with 1 × 10^13^ gc/kg of AAV‐TBG‐GFP (AAV‐GFP) vector. Following vector administrations, mice injected with AAV‐OAT showed significantly lower plasma ornithine concentrations compared with mice injected with the control vector expressing GFP (Fig EV3A). *Oat* ^*rhg*^ mice had a significant weight gain between 6 and 10 weeks of life and thus, vector loss due to liver growth might explain the lack of normalization of plasma ornithine concentrations (Fig EV3B). Compared with the eyes of control *Oat* ^*rhg*^ mice injected i.v. with the AAV‐GFP vector, retinal electrical activity by ERG showed that both the a‐ and b‐wave amplitudes significantly improved in the eyes of mice injected with AAV‐OAT vector that were comparable to wild‐type mice, suggesting that both photoreceptor (A wave) and bipolar cell (B wave) functions were preserved (Fig EV3C), because the A wave reflects hyperpolarization of photoreceptors, whereas the B wave arises from depolarization of bipolar cells and Müller cells. At 12‐months post‐injection, plasma ornithine was reduced, and lysine was increased in AAV‐OAT‐injected *Oat* ^*rhg*^ mice compared with AAV‐GFP‐injected controls (Fig EV3D) but no other changes in plasma amino acid concentrations were detected, except for a mild and clinically not relevant increase in glutamine (Table EV1). Moreover, ornithine concentrations were reduced in the eye cups of 6‐week‐old *Oat* ^*rhg*^ mice injected with AAV‐OAT compared with controls (Fig EV3E), thus supporting the hypothesis that retinal degeneration of GACR is dependent upon the systemic increase of blood ornithine concentration. Western blot on livers harvested at 12 months post‐injection showed greater OAT expression in mice injected with AAV‐OAT compared with mice injected with the control vector (Fig EV3F). Consistent with the ERG results, the retinal structure evaluated by H&E at 12 months post‐injection in *Oat* ^*rhg*^ mice injected with AAV‐OAT was comparable with unaffected wild‐type controls and strikingly different from *Oat* ^*rhg*^ mice injected with AAV‐GFP vector that showed abnormal retinal pigment epithelium (RPE) cells with irregular size and shape and disorganized photoreceptors of the outer segment layer (Fig EV3G).

**Figure 1:**
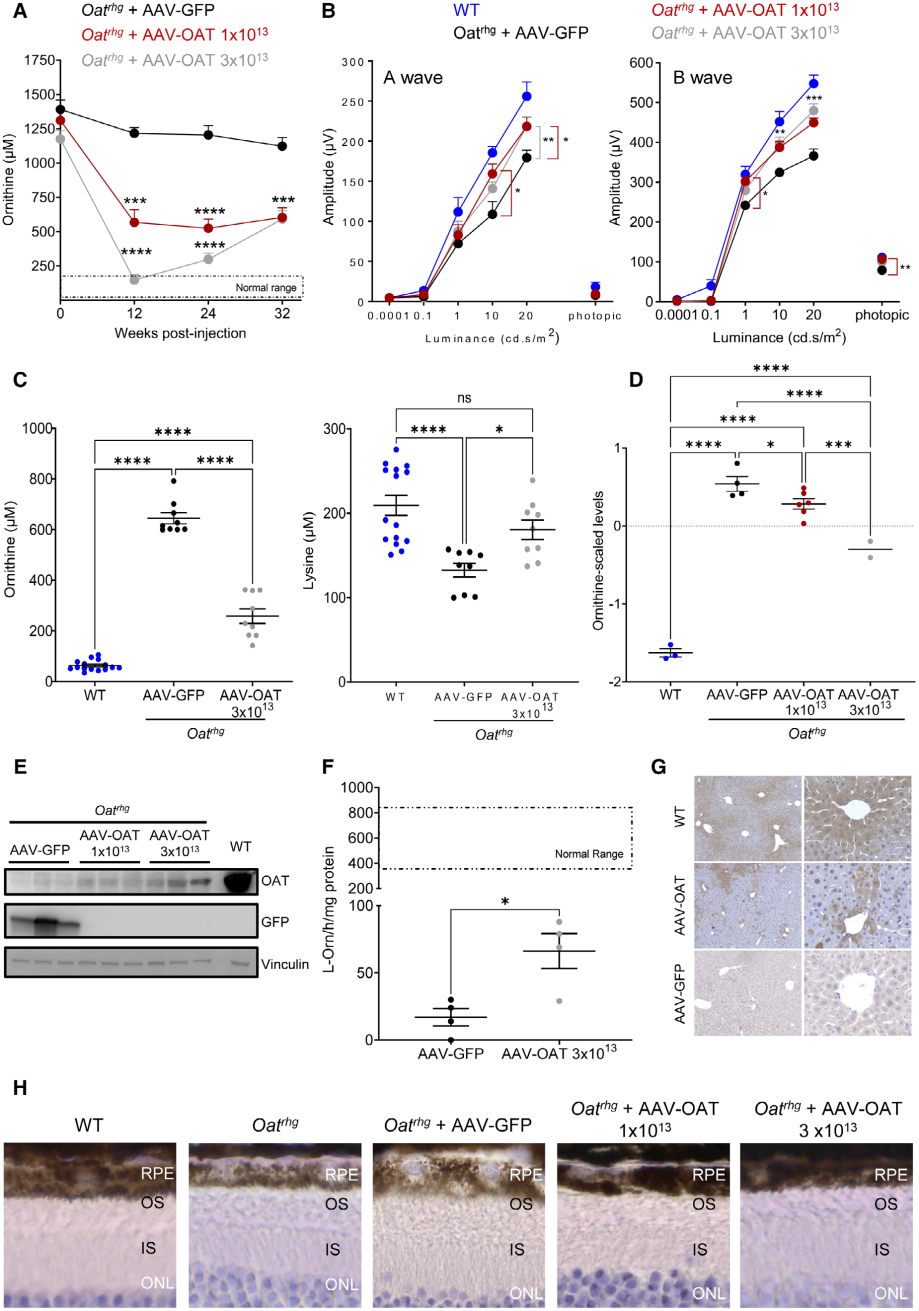
Improvement in retinal disease of *Oat* ^*rhg*^ mice by liver‐directed gene transfer Plasma ornithine concentrations in *Oat* ^*rhg*^ mice following intravenous injections of AAV‐OAT at the doses of 1 × 10^13^ (red circles, *n* = 9) and 3 × 10^13^ genome copies (gc)/kg (gray circles, *n* = 6), or AAV‐GFP at the dose of 1 × 10^13^ gc/Kg (black circles, *n* = 9). Normal range was established on wild‐type (WT) mice (*n* = 7) and average ± 2SD was calculated. Data are shown as averages ± SEM. ***P < 0.001, ****P < 0.0001 (Two‐way ANOVA test).Amplitude of a‐ and b‐waves under scotopic and photopic conditions (mean ± SEM) in uninjected WT mice (blue circles, *n* = 10), *Oat* ^*rhg*^ mice injected with AVV‐OAT of 1 × 10^13^ gc/kg (red circles, *n* = 12) or 3 × 10^13^ (gray circles, *n* = 6) and *Oat* ^*rhg*^ mice injected with AVV‐GFP (black circles, *n* = 8) at 11 months of age (9‐months post‐injection). Two‐way ANOVA test; *P < 0.05; **P < 0.01.Plasma ornithine and lysine concentrations at 12 months of age (10‐months post‐injection) in *Oat* ^*rhg*^ mice injected with AAV‐OAT (*n* = 3) or AAV‐GFP (*n* = 3); WT mouse controls (*n* = 5) are also shown; each sample was analyzed and shown as technical triplicates, data are shown as averages ± SEM. One‐way ANOVA test with Tukey correction; *P < 0.05; ****P < 0.0001; ns not statistically significant difference.Ornithine concentrations in the eye cups uninjected WT mice (*n* = 3), *Oat^rhg^* mice injected with AVV‐GFP (*n* = 4), *Oat^rhg^* mice injected with AVV‐OAT at the dose of 1 × 10^13^ gc/kg (*n* = 6) or 3 × 10^13^ (*n* = 2) at 12 months of age (10‐months post‐injection). Data are shown as *Z*‐scores; one‐way ANOVA with False Discovery Rate correction was used for comparison between groups. Means ± SEM are shown; *P < 0.05 ***P < 0.001, ****P < 0.0001.Western blot for OAT and GFP in livers of *Oat* ^*rhg*^ mice injected with AAV‐GFP or AAV‐OAT (doses are shown as gc/kg). A WT mouse liver was included as control. Vinculin was used as loading control.OAT activity on liver lysates of *Oat* ^*rhg*^ mice injected with AAV‐OAT (dose is shown as gc/kg) or AAV‐GFP vectors (*n* = 4 for each group). Data are shown as means ± SEM. Unpaired *t*‐test; *P < 0.05.Liver immunohistochemistry showing approximately 10% of OAT‐positive cells in *Oat* ^*rhg*^ mice injected with AAV‐OAT. Livers of WT and *Oat* ^*rhg*^ mice injected with AAV‐GFP are shown as controls. 5× (images on the left side) and 20× (images on the right side) fields of view are shown.Representative H&E staining of the retinas of 12‐month‐old *Oat* ^*rhg*^ mice injected with AAV‐GFP or AVV‐OAT at the doses of 1 × 10^13^ or 3 × 10^13^ gc/kg; age‐matched WT mice and uninjected *Oat* ^*rhg*^ mice were included as controls (40×). Abbreviations: RPE, retinal pigment epithelium; OS, outer segment; IS, inner segment; ONL, outer nuclear layer. Source data are available online for this figure.

**Figure 2:**
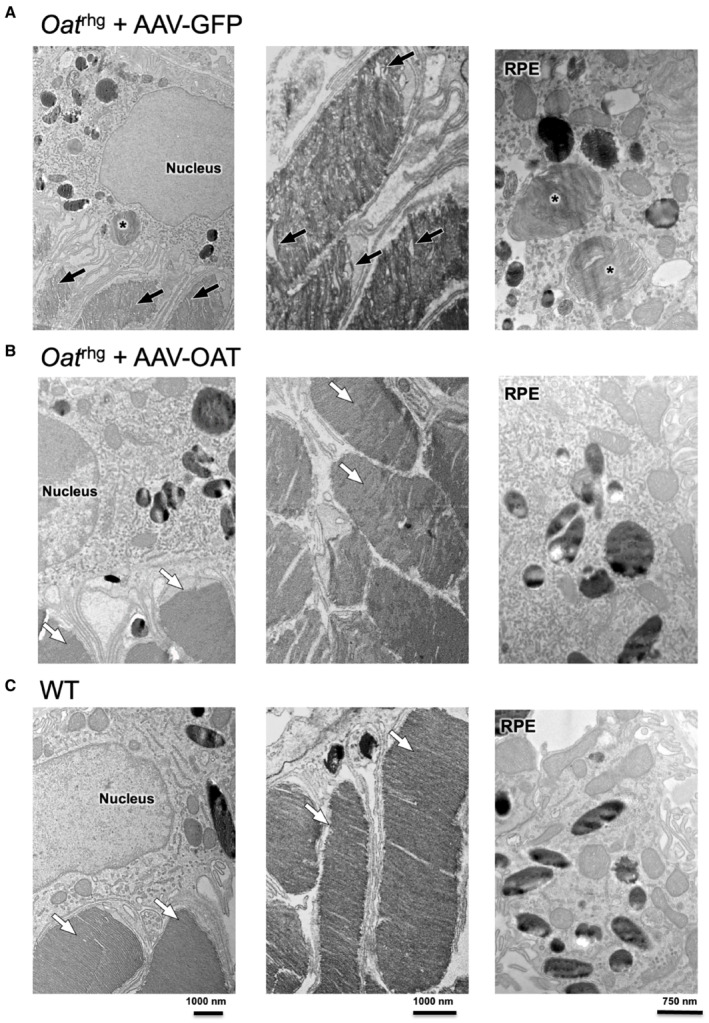
Ultrastructure of photoreceptor outer segment and retinal pigmented epithelium of *Oat* ^*rhg*^ mice after liver‐directed gene transfer of OAT Representative electron micrographs of the mouse retina of an AAV‐GFP injected *Oat* ^*rhg*^ mouse showing retinal pigmented epithelium (RPE) and adjacent abnormal outer segment (OS) disk membranes (black arrows). Phagolysosomes containing undigested OS disc membranes are present (asterisks).Retina of AAV‐OAT injected *Oat* ^*rhg*^ mice showing photoreceptor disk membranes (white arrows) and RPE ultrastructure comparable to age matched wild‐type (WT) mice (C, white arrows).Control retina of WT mouse is shown as comparison. Source data are available online for this figure.

**Figure EV4:**
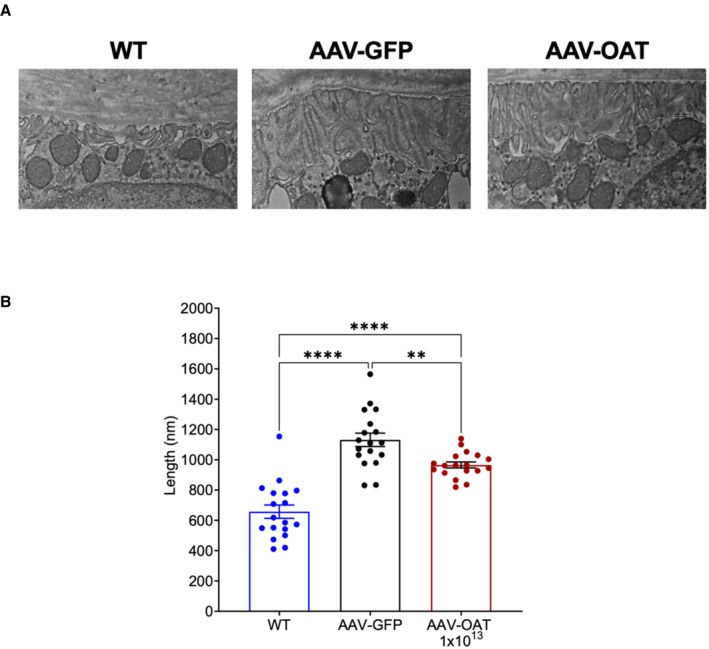
Electron microscopy of basal infoldings of Bruch's membrane of *Oat* ^*rhg*^ mice AAV‐GFP eyes (middle panel) displaying irregular and highly altered thickened ultrastructure of basal infoldings compared with wild‐type (WT‐left panel) or AAV‐OAT (dose is shown as gc/kg) injected mice (right panel). Basal infoldings of Bruch's membrane is outlined by dashed line. Scale bar: 750 nm.Quantification of basal membrane thickness. Layers' depth was measured and averaged from six fields for each group, three measurements for each image. One mouse was analyzed for each group. One‐way ANOVA test with Tukey correction was performed to compare experimental groups. Means ± SEM are shown **P < 0.01; ****P < 0.0001.

To overcome the vector dilution due to the delayed growth, *Oat* ^*rhg*^ mice were injected at 10 weeks of age when the mouse weight reached the adult size, and they showed a greater approximately 60% reduction in baseline plasma ornithine concentrations after the injection of 1 × 10^13^ gc/kg of AAV‐OAT vector (Fig 1A). Further reduction in plasma ornithine concentrations was observed in mice injected with the higher dose of 3 × 10^13^ gc/kg (Fig 1A). By ERG, compared with mice injected with AAV‐GFP, eyes of *Oat* ^*rhg*^ mice treated with both doses of AAV‐OAT showed a significant improvement of both a‐ and b‐wave amplitudes at high luminance intensities in flash dark‐adapted scotopic and photopic condition at 11 months post‐injection (Fig 1B). At the time of sacrifice 12‐months after gene therapy, *Oat* ^*rhg*^ mice injected with the AAV‐OAT vector were confirmed to have increased plasma concentrations of lysine and reduced plasma ornithine concentrations (Fig 1C), but no other relevant changes in plasma amino acid concentrations compared with *Oat* ^*rhg*^ mice injected with AAV‐GFP or wild‐type control mice (Table 1). Moreover, ornithine concentrations were also reduced in the eye cups (Fig 1D). OAT protein expression and activity were increased in livers of *Oat* ^*rhg*^ mice injected with AAV‐OAT compared with control mice (Fig 1E and F) and OAT‐positive hepatocytes were detected by liver immunohistochemistry (Fig 1G). At 12 months post‐injection, the retinal structure of *Oat* ^*rhg*^ mice injected with AAV‐GFP vector showed retinal degeneration with irregular swelling and frequent doming in the RPE (Fig 1H). In contrast, the RPE of *Oat* ^*rhg*^ mice injected with AAV‐OAT at both doses showed a compacted appearance similar to wild‐type mice (Fig 1H). By transmission electron microscopy (TEM), *Oat* ^*rhg*^ mice injected with AAV‐GFP showed disorientated and swollen photoreceptors with disorganized disc membranes and frequent phagolysosomes containing undigested material (Fig 2A), whereas mice injected with AAV‐OAT exhibited near normal ultrastructure (Fig 2B) similar to wild‐type controls (Fig 2C). Moreover, basal infoldings of the Bruch's membrane *Oat* ^*rhg*^ mice injected with AAV‐GFP was irregularly thickened with discontinuous layers compared with wild‐type mice (Fig EV4). In contrast, layer thickness was reduced in *Oat* ^*rhg*^ mice injected with the AAV‐OAT vector (Fig EV4).

**Table 1:** Plasma amino acids (μM) in *Oat* ^*rhg*^ mice injected with AAV‐OAT or AAV‐GFP at 12‐months post‐injection. Wild‐type (WT) control mice are also shown.

Plasma A.A. (μM)	WT		AAV‐OAT		AAV‐GFP	
	Average	SD	Average	SD	Average	SD
ASP	11.4	9.8	5.7	2.7	2.9^a^	1.9
GLU	57.3	19.0	51.9	7.1	33.7^a^ ^,^ ^b^	10.6
ASN	60.6	24.6	34.2^a^	9.8	28.9^a^	3.1
SER	107.1	16.0	81.2^a^	22.2	76.7^a^	7.9
GLN	662.3	37.0	437.9^a^	80.0	410.5^a^	44.0
HIS	120.1	40.2	156.6	84.0	79.7^a^	11.7
GLY	207.7	37.1	293.8^a^	10.4	167.4^a^ ^,^ ^b^	33.4
THR	152.1	35.6	110.5^a^	17.5	113.1^a^	20.3
CIT	60.2	19.0	97.3	51.6	42.6^b^	18.9
ARG	71.4	34.0	70.2	4.0	62.8	7.5
ALA	366.1	80.6	356.7	29.3	283.4^a^ ^,^ ^b^	47.6
TYR	69.8	25.3	52.0	16.4	46.5^a^	3.7
VAL	172.7	33.1	123.0^a^	6.2	134.2^a^	25.5
MET	60.5	15.5	58.2	14.2	43.3^a^	11.1
TRP	24.9	16.5	12.4^a^	2.6	9.1^a^	5.3
PHE	78.5	16.3	66.2	10.2	48.1^a^ ^,^ ^b^	7.3
ILE	76.6	10.3	46.7^a^	5.4	57.2^a^ ^,^ ^b^	9.7
LEU	123.7	22.6	91.4^a^	17.6	92.5^a^	15.2
PRO	98.5	29.7	85.6	21.8	65.1^a^	6.2

^a^ Mean value significantly different from WT (*P* < 0.05).

^b^ AAV‐GFP mean value significantly different from AAV‐OAT (*P* < 0.05).

**Figure EV5:**
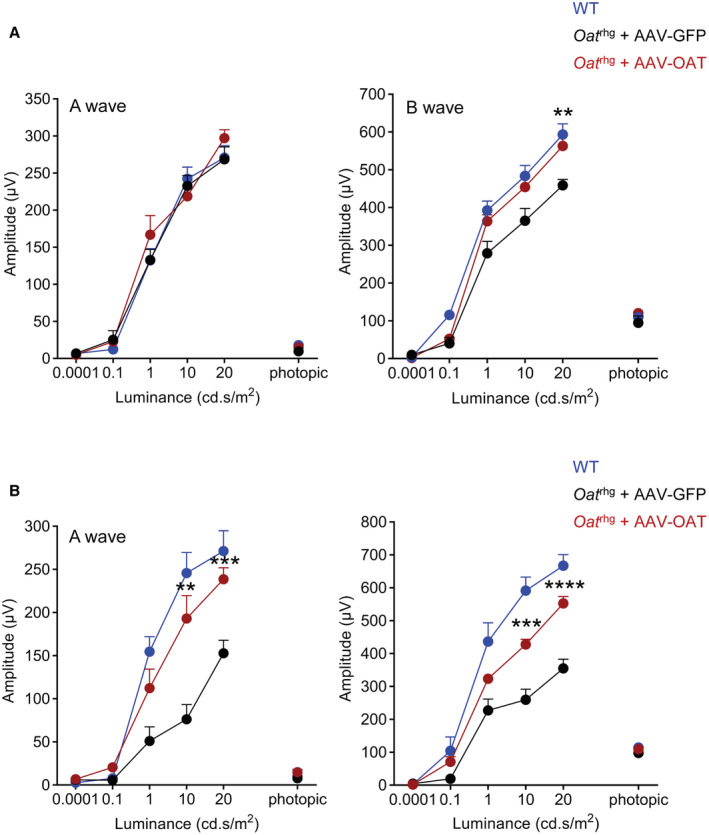
AAV‐mediated liver‐directed OAT delivery into *Oat* ^*rhg*^ albino mice A, BERG components (a‐ and b‐ waves) under scotopic and photopic conditions of 7‐month‐old (A) and 11‐month‐old (B) *Oat* ^*rhg*^ albino mice injected with 1 x 10^13^ gc/kg of AAV‐OAT (*n* = 10, 7‐month‐old; *n* = 7, 11‐month‐old) or AAV‐GFP vectors (*n* = 6, 7‐month‐old; *n* = 7, 11‐month‐old). Wild‐type (WT) mice were included as control (*n* = 7, 7‐month‐old; *n* = 3, 11‐month‐old). All data are shown as averages ± SEM. Two‐way ANOVA test was used for comparison between groups. **P < 0.01; ***P < 0.001, ****P < 0.0001.

The *rhg* mutation arose spontaneously in the AKR/J genetic background, that were later outcrossed to C57BL/6JEi for line maintenance. As AKR/J mice are an albino strain, by crossing *Oat* ^*rhg*^ mice, mice with white fur are also obtained. As expected, these *Oat* ^*rhg*^ white mice showed signs of ERG abnormalities at earlier age compared with the black mice and showed an improved ERG response following the i.v. injections performed at 10 weeks of age with AAV‐OAT compared with control mice injected with AAV‐GFP (Fig EV5).

**Figure 3:**
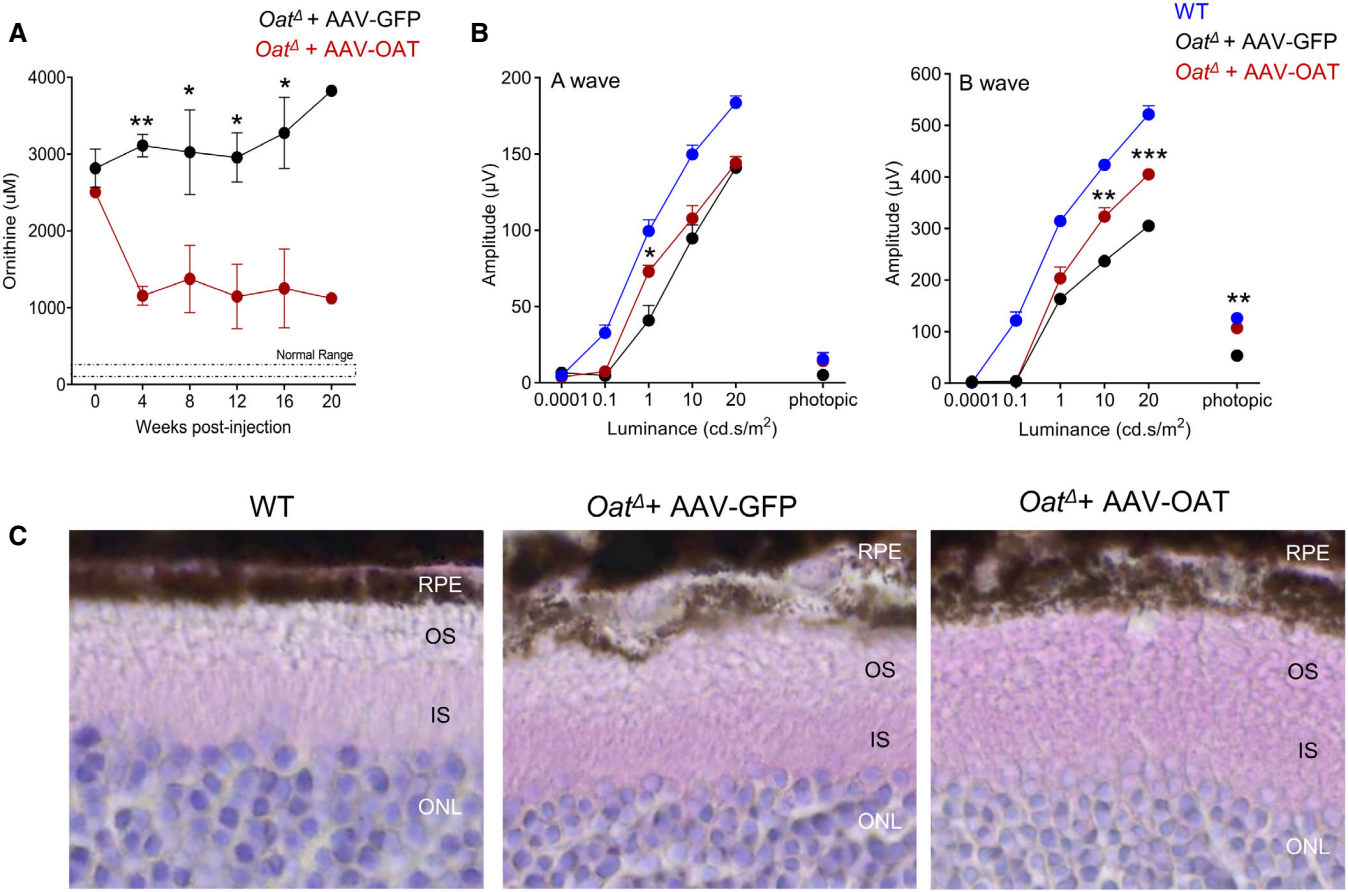
Improvement phenotype in *Oat* ^*Δ*^ mice following AAV‐mediated liver‐directed OAT gene transfer Plasma ornithine concentrations in AAV‐GFP‐ (black circles, *n* = 3) and AAV‐OAT‐injected (red circles, *n* = 3) at the dose of 3 x 10^13^ gc/kg in *Oat* ^*Δ*^ mice. Normal range of wild‐type (WT) mice is also shown. Each value plotted represents mean ± SEM; two‐way ANOVA test, *P < 0.05, **P < 0.01.ERG at six months post‐injection in *Oat* ^*Δ*^ mice injected with either AAV‐GFP (black circles, *n* = 6) or AVV‐OAT (red circles, *n* = 5); WT controls are also shown (blue circles, *n* = 5). Two‐way ANOVA; test *P < 0.05; **P < 0.01; ***P < 0.001. Data are shown as averages ± SEM.Retinal H&E (40×) of *Oat* ^*Δ*^ mice injected with either AAV‐GFP or AAV‐OAT. Representative of three mice per groups are shown; WT mice were included as control. Abbreviations: RPE, retinal pigment epithelium; OS, outer segment; IS, inner segment; ONL, outer nuclear layer. Source data are available online for this figure.

Oat *null* mice (*Oat* ^*Δ*^) have increased perinatal mortality and die 24–48 h after birth if not supplemented by intraperitoneal injections of arginine to prevent hyperammonemia (Wang *et al*, 1995, 1996). Compared with *Oat* ^*rhg*^ mice, *Oat* ^*Δ*^ mice have earlier onset of retinal degeneration and show signs of loss of retinal function by 4 months of age (Wang *et al*, 2000). Consistent with the results in the *Oat* ^*rhg*^ mice, 6‐week‐old *Oat* ^*Δ*^ mice injected with 3 × 10^13^ gc/kg of AAV‐OAT showed sustained reduction in plasma ornithine concentrations (Fig 3A) and improvement in ERG response and retinal pathology (Fig 3B and C).

## Discussion

GACR is a severe and progressive form of blindness caused by deficiency of OAT that results in a systemic increase in ornithine concentrations. The only available treatment is a stringent diet that suffers of poor adherence. In the present study, we showed that liver‐directed gene transfer of the OAT gene by an AAV vector restored the activity of the enzyme in the liver, reduced ornithine concentrations in plasma and in eye cups, and improved retinal function and structure in two independent GACR mouse models. Preclinical studies in OAT‐deficient mice have previously shown that low‐arginine diet reducing plasma ornithine concentrations, delay retinal degeneration. Therefore, it was hypothesized that systemic correction of the defect may prevent retinal disease (Wang *et al*, 2000). This hypothesis is supported by the reduced ornithine concentration in eye cups as consequence of liver‐specific expression of OAT shown in the present study.

While no differences in reductions of ornithinemia between albino or pigmented *Oat* ^*rhg*^ mice were detected following injections of the AAV‐OAT vector, *Oat* ^*rhg*^ mice with white fur showed earlier impairment of b‐waves on ERG by 7 months of age that was corrected by AAV‐OAT injections. Therefore, albino *Oat* ^*rhg*^ mice might be used to detect therapeutic efficacy at earlier time points compared with pigmented mice.

The results of the present study also suggest that complete normalization of blood ornithine might not be necessary to prevent the retinal degeneration, and concentrations below 1,000 μM might be sufficient to prevent the retinal degeneration. This threshold is consistent with previous studies suggesting that blood ornithine concentrations exceeding 600 μM induce retinal toxicity (Hayasaka *et al*, 2011). Moreover, this study suggests that OAT activity in liver does not need to be normalized to improve retinal structure and function and approximately 10% of activity might be sufficient to achieve clinical benefit. These therapeutic thresholds make GACR an attractive disease candidate because phenotypic correction might be achieved with relatively lower vector dose.

Several genes exhibit a spatially graded expression pattern in hepatocytes and OAT is expressed at relatively low amounts, with a striking pericentral zonation (Halpern *et al*, 2017). Because of the metabolic zonation along the porto‐central axis, gene therapy should aim at expression of therapeutic proteins within the zone where they are physiologically localized. However, it has been found that AAV transduction is not uniform within the hepatic lobule. In mice for example, AAV8 transduces predominantly hepatocytes near central veins and yields lower transduction levels in hepatocytes in the periportal regions. The pericentral dominance in transgene expression in mice injected with AAV8 is observed with both liver‐specific and ubiquitous promoters, indicating that transduction zonation is not caused by promoter specificity (Bell *et al*, 2011). In contrast to mice, in nonhuman primates the expression pattern from AAV8 vectors is reversed, i.e., transgene expression is higher around portal areas and less intense or absent around central veins (Bell *et al*, 2011). Therefore, AAV8 might not be the best suited AAV vector serotype for human applications and AAV vectors with a preferential pattern of pericentral hepatocytes in humans should be preferred. While OAT expression is pericentral, urea cycle enzymes are expressed periportally (Halpern *et al*, 2017). Therefore, liver expression of OAT in pericentral hepatocytes might result in depletion of ornithine that is removed from the urea cycle to be converted into P5C, thus resulting in defective ureagenesis. Compared to AAV‐GFP‐injected mice, plasma glutamine was slighlty higher in AAV‐OAT‐injected *Oat* ^*rhg*^ mice but the concentration was lower than wild‐type controls. Moreover, no reduced citrulline was detected, thus excluding an impairment of ureagenesis. Nevertheless, a defect in ureagenesis remains possible under conditions of high OAT expression. In case of supraphysiologic OAT activity, potential risks of cancer should also be considered because OAT has been found to be overexpressed in hepatocellular carcinoma (Colnot *et al*, 2004) and *in vivo* inhibition of OAT has an antitumor effect in mice (Zigmond *et al*, 2015).

This study also suggests that ornithine plays an important role in the retinal disease. Interestingly, ornithine toxicity appears to be specific for cells of the RPE (Ando *et al*, 2000). OAT catalyzes the reversible conversion of ornithine to P5C, which can be further metabolized to proline and glutamate. Depending on the tissue, OAT catalyze the reaction either of ornithine degradation or synthesis (Ginguay *et al*, 2017). Therefore, the mechanism underlying the retinal degeneration might be either ornithine accumulation or deficiency of ornithine or its downstream metabolites, such as proline. Studies on human cultured RPE cells showed that inhibition of ornithine intracellular transport reduced cell toxicity, supporting a direct toxicity of excessive ornithine (Nakauchi *et al*, 2003). Consistently, spermine, an ornithine metabolite, was found to induce apoptosis of RPE cells (Kaneko *et al*, 2007). Moreover, retinal degeneration has been anecdotally reported in patients with hyperornithinemia–hyperammonemia–homocitrullinuria syndrome (HHH syndrome) that have increased ornithine concentrations in blood despite normal OAT activity (Lemay *et al*, 1992).

AAV‐mediated liver‐directed gene therapy is starting to generate promising results in clinical trials for hemophilia and inherited metabolic disorders (Nathwani *et al*, 2011; Brunetti‐Pierri *et al*, 2022). Based on these encouraging results, an increasing number of disease targets are under consideration for future clinical trials and GACR appears an attractive candidate. The availability of an excellent disease‐marker correlating with the disease, i.e., plasma ornithine further support GACR as a gene therapy candidate. However, the lack of studies on natural history of the disease and the best timing for intervention need to be addressed. Nevertheless, GACR will need intervention prior to the onset of the retinal degeneration. Importantly, GACR can be potentially diagnosed pre‐symptomatically by newborn screening (de Sain‐van der Velden *et al*, 2012), thus allowing early intervention prior to the onset of the retinal damage. The reversed enzyme reaction catalyzed by OAT in early infancy results in low ornithine and citrulline concentrations and increased proline. Consequently, the proline/citrulline ratio in plasma is increased in GACR patients compared with controls and applying this ratio for newborn screening could lead to early and specific detection of neonatal OAT deficiency, with no additional expense to newborn screening laboratories quantifying amino acids (de Sain‐van der Velden *et al*, 2012).

## Materials and Methods

### Generation of AAV vectors

The human OAT coding sequence was obtained by OriGene Technologies Inc. (Rockville) and amplified by PCR with the following primers: Forward—5′‐ATAAGAATGCGGCCGCATGTTTTCCAAACTAGC‐3′; Reverse—5′‐CCCAAGCTTTCAGAAAGACAAGATGG‐3′. PCRs were performed using Phusion High‐Fidelity DNA Polymerase (NEB, M0530) and PCR products were then digested with *Not*I and *Hind*III, and subcloned into a pAAV2/8.TBG.GFP plasmid (Auricchio *et al*, 2001) by removing the GFP coding sequence (*Not*I–*Hind*III). The same pAAV2/8.TBG.GFP was used for production of the control vector. AAV vectors were produced by Innovavector s.r.l., Italy by triple transfection in HEK293 cells, purified by CsCl_2_ ultracentrifugation, and titered (in genome copies/milliliter) by real‐time PCR‐based assay and a dot blot analysis.

### Mice and procedures

The study was authorized by the Italian Ministry of Health (approval number 860). *Oat* ^*rhg*^ mice were purchased from the Jackson Laboratory (JAX stock #003544) and housed at TIGEM animal facility. Animals were inbred and maintained under normal animal house conditions: they were kept in ventilated cages in a 12‐h light–dark cycle environment, they received a standard chow diet and water *ad libitum*. To specifically identify the G to C transversion in the *Oat* gene the following primers flanking exon 9 of the *Oat* gene were used: Forward—5′ AGGCCCTGAAAATGAAAAGCATAC‐3′ and Reverse—5′ ‐GAGCTGCCTAACTAACAAAGACAG‐3′ that generated a 499 bp amplimer by PCR from mutant, heterozygous, and wild‐type mice. The purified amplimers were digested with *Hae*III, electrophoresed, and visualized on 3% agarose gel. Mutant *rhg*‐associated *Oat* amplimers were cut with *Hae*III in four bands (185 bp, 166 bp, 164 bp, 42 bp), whereas wild‐type amplimers were cut in three bands (351 bp, 164 bp, 42 bp). *Oat* ^*Δ*^ mice were kindly provided by Dr. David Valle, The Johns Hopkins University School of Medicine, Baltimore, MD, USA. Homozygous *Oat* ^*Δ*^ mice were obtained by heterozygote mating and at birth they were administered with arginine by intraperitoneal injection at 12‐h intervals up to 3–14‐days of age, as previously described (Wang *et al*, 1995). For genotyping of *Oat* ^*Δ*^ mice, the following primers were used for PCR: Forward‐1—5′‐AACTAGCAAGTCTGCAGACC‐3′ and Reverse—5′‐TCCACAAGGCATTCAGTGCG‐3′ designed on the wild‐type allele to generate a 280 bp amplimer; Primer pair Forward‐2—5′‐CTGAGGCGGAAAGAACCAG‐3′ and the same reverse to yield a 220 bp amplimer from *Oat* ^*Δ*^ mouse DNA. The i.v. injections of AAV vectors were performed in a final volume of 200 μl into retro‐orbital plexus of 6‐ or 10‐week‐old mice. Both male and female mice were used. No statistical methods were used for sample size calculation. To measure plasma ornithine concentrations, blood samples were collected by submandibular bleedings at baseline, and after the injections. Body weights were also evaluated. Treated animals were sacrificed by sub‐lethal injection of ketamine/medetomidine (300 and 10 mg/kg, respectively) and perfused with ice‐cold phosphate‐buffered saline (PBS) at 12 months of age; livers and eyes were harvested for further analyses. Wild‐type littermates were used as controls. For all studies, wild‐type and affected mice were housed under the same condition. Experiments were not performed in a blinded fashion.

### Metabolite measurements

Plasma ornithine concentrations were monitored on samples collected at various times post‐vector administration using an ornithine fluorometric assay kit (Abcam; Cat# ab252903) according to the manufacturer's instructions. Briefly, in this enzyme‐based assay, ornithine is converted into a series of intermediates, reacting with a probe producing a stable fluorometric signal (Ex/Em = 535/587 nm). At sacrifice plasma amino acid concentrations were measured by HPLC using an Agilent Technologies 1,200 Series LC System with an Agilent Zorbax Eclipse XDB‐C18 analytical column (5 μm, 4.6 × 150 mm) and Agilent Eclipse XDB‐C18 analytical guard column (5 μm, 4.6 × 12.5 mm). Aliquots of 500 μl of plasma were analyzed. Automated and online derivatization using o‐phthalaldehyde (OPA) for and 9‐fluorenylmethyl chloroformate (FMOC) was carried out for primary and secondary amino acids, respectively. Amino acids were separated by elution at a flow rate of 1.3 ml/min at 40°C with a linear gradient of solvent B (CH_3_CN/CH_3_OH/H_2_O, 40/40/20) in solvent A (40 mM Phosphate buffer pH 7.8) from 10 to 20% in 6 min, from 20 to 27% in 6 min, from 27 to 60% in 10 min, from 60 to 100% in 2 min, followed by isocratic step to 100% for 6 min. Amino acids were identified by their retention time and quantified by absorption ratio by comparison with the ratio of authentic compounds in the calibration solution, composed by a mixture of amino acids in final concentration of 200 μM. Murine eye cups for ornithine determination were harvested upon mice sacrifice and perfusion, dissected under a light microscope to isolate the eye cups from lens and immediately frozen in liquid nitrogen. Collected samples were processed as previously described (Audano *et al*, 2021) and ornithine concentrations were obtained by liquid chromatography coupled to tandem mass spectrometry.

### Electrophysiological analyses

Mice were dark adapted for 180 min and anesthetized with an intraperitoneal injection of ketamine/medetomidine (100 and 0.25 mg/kg, respectively); their pupils were dilated with a drop of 1% tropicamide (Visufarma, Rome, Italy), and body temperature maintained at 37.5°C. Flash electroretinograms were recorded by gold‐plate electrodes positioned on the cornea of each eye, referred to a needle electrode inserted subcutaneously in the frontal region. The different electrodes were connected to a two‐channel amplifier. Scotopic ERG were evoked by light flashes increasing (0.0001; 0.1; 1; 10; 20 cd.s/m^2^) in dark conditions. The time interval between stimuli is 5 s for the first three luminance and 60 between the luminance 10 and 20 cd.s/m^2^. Photopic ERG were recorded after the scotopic session by stimulating the eye with 10 flashes of 20.0 cd.s/m^2^ over a constant background illumination of 50 cd/m^2^. A‐ and B‐waves were plotted as a function of increasing light intensities.

### Retina pathology and ultrastructure

Eyes from 12‐month‐old *Oat* ^*rhg*^ and 8‐month‐old *Oat* ^*Δ*^ mice were harvested and fixed by immersion in Davidson's Fixative (acetic acid, Ethanol, and formaldehyde). Before harvesting, temporal side of the sclera was marked with a suture stitch (Vicryl 7‐0, ethicon) to allow eye orientation in paraffin. After 24 h, samples were dehydrated in 70% Ethanol and paraffin embedded. Seven μm‐thick sections were transversally cut on a Leica RM2125 RTS microtome and progressively distributed on 10 slides to have 15–21 sections representative of the whole eye at different levels in each slide. Sections were stained with hematoxylin and eosin and stained section were examined by light microscopy. Images were captured using ZEISS Axioscan 7 Microscope Slide Scanner.

For TEM, murine eyes were collected upon sacrifice by sub‐lethal injection of ketamine/medetomidine (300 and 10 mg/kg, respectively) and perfusion with a mixture of 2% paraformaldehyde and 1% glutaraldehyde prepared in 0.2 M HEPES buffer (pH 7.4). The eye was gently secured and lens and iris were removed though the opening made by cornea excision. The optic cup was centered at the base of the eye and bisected parallel to the median plane; then this half‐sphere was bisected again with each of the two cuts. Samples were placed on a slow rotator to circulate the fixative for 30 min and stored in PBS at 2°C to 8°C. Next, samples were postfixed, dehydrated and embedded in epoxy resin, as described previously (Polishchuk & Polishchuk, 2019). 60‐nm thin sections were cut on a Leica EM UC7 microtome. TEM images were acquired from thin sections using a FEI Tecnai‐12 electron microscope equipped with a VELETTA CCD digital camera (FEI, Eindhoven, the Netherlands). For basal infoldings of Bruch's membrane quantification, six images from each experimental group were acquired and analyzed at the same magnification. From each image, three measurements of basal infoldings of Bruch's membrane in RPE were taken. Each measurement was considered as *n* = 1, and thus a total of *n* = 18 was collected. iTEM software was used for thickness quantification.

### Immunohistochemistry and immunofluorescence

Mouse liver samples were fixed in 4% paraformaldehyde overnight, stored in 70% ethanol after washing in PBS 1×, and finally embedded into paraffin blocks. 5‐μm thick sections were rehydrated and permeabilized in PBS with 0.5% Triton (Sigma‐Aldrich) for 20 min. Antigen unmasking was performed in 0.01 M citrate buffer pH 6.0 in a microwave oven. Next, sections underwent blocking of endogenous peroxidase activity in methanol/1.5% H_2_O_2_ (Sigma‐Aldrich) for 30 min and were incubated in blocking solution (3% BSA [Sigma‐Aldrich], 5% donkey serum [Millipore], 1.5% horse serum [Vector Laboratories] 20 mM MgCl_2_, 0.3% Triton [Sigma‐Aldrich] in PBS) for 1 h. Sections were stained with rabbit anti‐OAT primary antibody (Abcam; Cat# ab137679, dilution 1:100) overnight at 4°C and with universal biotinylated horse anti‐mouse IgG secondary antibody (dilution 1/200) (Vector Laboratories, PK‐620) for 1 h RT. Biotin/avidin‐horseradish peroxidase (HRP) signal amplification was achieved using ABC Elite Kit (Vector Laboratories, PK‐6200) according to the manufacturer's instructions. 3,3′‐diaminobenzidine (Vector Laboratories) was used as peroxidase substrate. Mayer's hematoxylin (Bio‐Optica) was used as counter‐staining. Sections were de‐hydrated and mounted in VECTASHIELD® (Vector Laboratories). Image capture was performed using an Axioscan microscope (Zeiss).

Immunofluorescence staining was performed on paraffin sections of livers. Liver samples were fixed in 4% PFA overnight, washed three times with PBS and stored in 70% Ethanol. Finally, they were embedded into paraffin blocks and cut into 5 μm sections. The sections were rehydrated and permeabilized for 20 min in PBS containing 0.2% Triton® X‐100. Blocking solution containing 5% donkey serum was applied for 1 h. Primary antibodies were diluted in blocking solution and incubated overnight at 4°C. Anti‐GFP (Abcam, Cat# ab13970) was used at 1:900 dilution and rabbit anti‐OAT (Abcam; Cat# ab137679) was used at 1:200 dilution. Next, sections were washed three times in PBS for 5 min each and then incubated with fluorescently labeled secondary antibodies. The secondary antibody Anti‐chicken Fluor® 488, and anti‐rabbit Alexa Fluor® 594 were used at 1:400 dilutions. Sections were washed three times in PBS for 5 min each and mounted on glass slides with Vectashield (Vector Laboratories, Burlingame, CA) mounting reagent. Images were obtained using ZEISS Axioscan 7 Microscope Slide Scanner.

### Western blot and enzyme activity

Liver specimens were mechanically homogenized using metal beads and lysed in RIPA buffer, supplemented with protease inhibitor cocktail (Sigma). Samples were incubated for 30 min on ice, vortexed every 10 min, and centrifuged at 16,200 *g* for 20 min. Pellets were discarded and lysates were used for Western blot analyses. After lysis, protein concentration was determined by Bradford Reagent (Bio‐Rad) and samples were denatured at 100°C for 5 min in 1× Laemmli sample buffer supplemented with 1 M dithiothreitol (DTT). Next, protein extracts were resolved on SDS–PAGE and transferred onto polyvinylidene difluoride (PVDF) membranes. Thirty micrograms of liver proteins were loaded for each specimen into a 4–15% SDS–PAGE; after transfer to PVDF membrane, blots were blocked with TBS‐Tween‐20 containing 5% non‐fat milk for 1 h at room temperature followed by incubation with primary antibody overnight at 4°C. The primary antibodies used for immuno‐blotting were: rabbit anti‐OAT (Abcam; Cat# ab137679; dilution: 1/1,000), mouse anti‐Calnexin (Santa Cruz Biotechnology; Cat# sc23954; dilution: 1/1,000), mouse anti‐Vinculin (Santa Cruz Biotechnology; Cat# sc73614; dilution: 1/1,000) and rabbit anti‐GFP (Novus Biologicals; Cat# nb 600‐308; dilution: 1/1,000). Proteins of interest were detected with HRP‐conjugated goat anti‐mouse or anti‐rabbit IgG antibody (GE Healthcare). Peroxidase substrate was provided by ECL Western Blotting Substrate kit (Pierce).

To measure OAT activity, liver specimens were lysed in PBS with protease inhibitor cocktail (Roche) by sonication (4 × 5 s, 10 s pause) on ice. Enzyme activity was determined by a spectrophotometric assay measuring the dihydroquinazolium derivative of P5C after incubation with 2‐aminobenzaldehyde as previously described (Montioli *et al*, 2017). Briefly, 100 μg of the soluble crude liver lysates were incubated in 50 mM Hepes, pH 8.0, 150 mM NaCl and 100 μM pyridoxal 5' phosphate at 25°C with saturating concentration of L‐Ornithine (100 mM) and α‐ketoglutarate (50 mM) for 45 min. Reactions were stopped by 10% (v/v) trichloroacetic acid and samples incubated for 40 min at 25°C with a fresh solution of 15 mM 2‐aminobenzaldehyde to allow color development. After centrifugation (12,000 *g* for 2 min), the absorbance at 440 nm of the supernatant was measured and a molar extinction coefficient of 2.71 × 10^3^ M^−1^ cm^−1^ was used to calculate the amount of P5C formed.

### Statistical analyses

Data were statistically analyzed using GraphPad Prism software. Two‐way ANOVA was used to show significance difference for mean comparisons of ERG data and plasma ornithine concentrations. Ordinary one‐way ANOVA was used to show significance differences in ornithine levels in eye cups and amino acid concentrations in plasma measured by HPLC, and basal infolding of Bruch's membrane. Unpaired *t*‐test was used for groups comparison of OAT enzyme activity. Experimental group sizes are reported in the figure legends. Data are shown as average ± SEM.

## Author contributions

**Iolanda Boffa:** Conceptualization; data curation; formal analysis; writing – original draft. **Elena Polishchuk:** Data curation. **Lucia De Stefano:** Data curation. **Fabio Dell'Aquila:** Data curation. **Edorado Nusco:** Data curation. **Elena Marrocco:** Data curation. **Matteo Audano:** Data curation. **Silvia Pedretti:** Data curation. **Marianna Caterino:** Data curation. **Ilaria Bellezza:** Data curation. **Margherita Ruoppolo:** Supervision. **Nico Mitro:** Supervision. **Barbara Cellini:** Supervision. **Alberto Auricchio:** Supervision. **Nicola Brunetti‐Pierri:** Conceptualization; data curation; formal analysis; supervision; funding acquisition; investigation; writing – original draft; writing – review and editing.

## Disclosure and competing interests statement

The authors declare that they have no conflict of interest.

## For more information

- https://omim.org/
- https://www1.rarediseasesnetwork.org/cms/ucdc
- https://rarediseases.info.nih.gov/diseases/6556/gyrate‐atrophy‐of‐choroid‐and‐retina

## Supporting information



Expanded View Figures PDF

Table EV1

PDF+

Source Data for Figure 1

Source Data for Figure 2

Source Data for Figure 3

Source Data for Table 1

Source Data for Table EV1

## Data Availability

This study includes no data deposited in external repositories.
