# Supplementary material for: Liver‐directed gene therapy for ornithine aminotransferase deficiency
Source: EMBO Mol Med. 2023 Jan 17;15(4):e17033. doi: 10.15252/emmm.202217033 (PMC10086579; doi:10.15252/emmm.202217033)
Supplement: Supplementary file 1 — Expanded View Figures PDF [file EMMM-15-e17033-s007.pdf]

Expanded View Figures

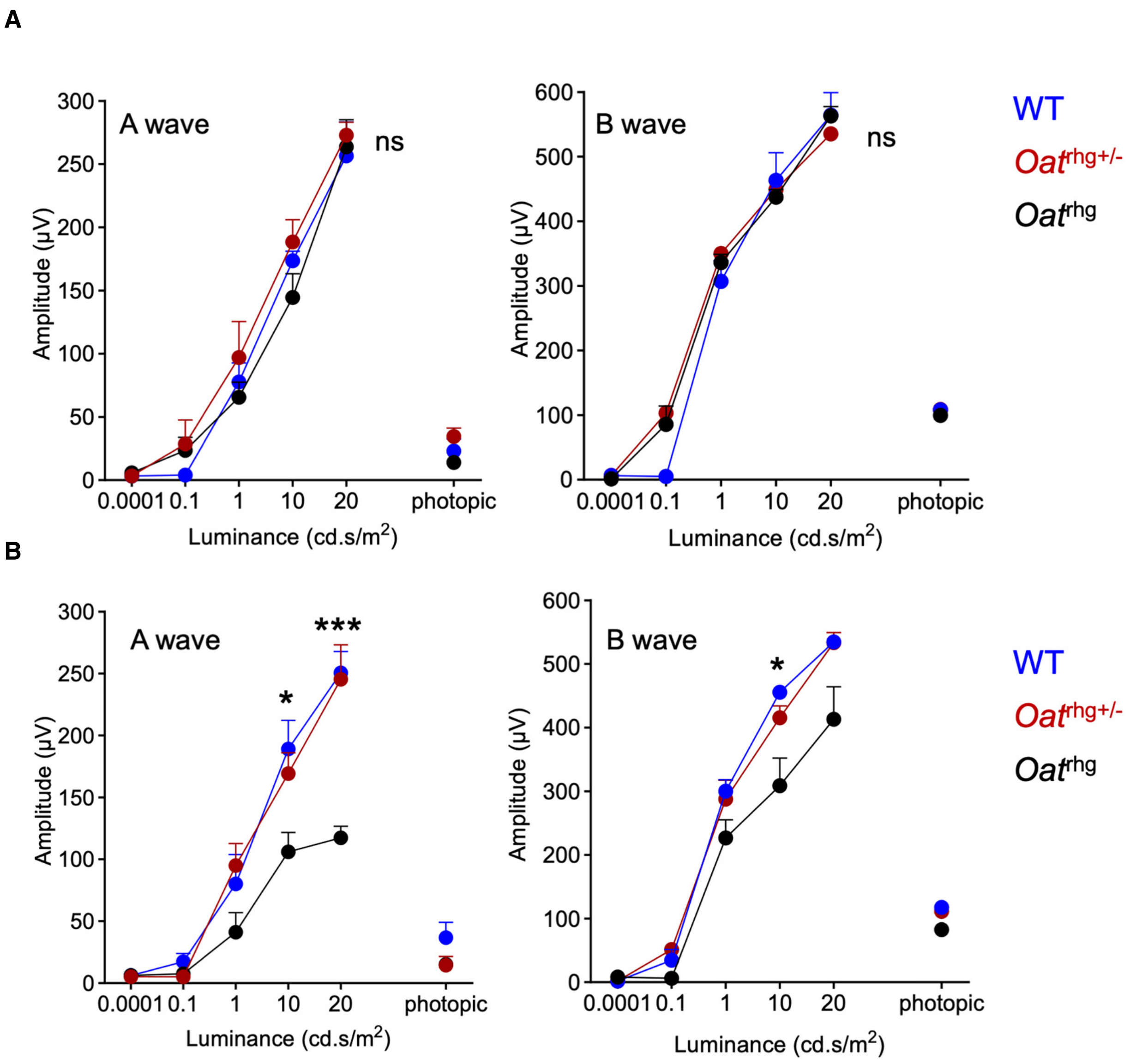

Figure EV1. Retinal function in *Oat*^{rhg} mice.

A, B a- and b-wave amplitudes under scotopic and photopic conditions in *Oat*^{rhg} pigmented mice. The amplitudes induced by increasing light intensities under scotopic conditions in 7-month-old (A) and 12-month-old (B) wild-type (blue circles, $n = 6$), heterozygous *Oat*$^{+/rhg}$ (red circles, $n = 6$) and homozygous *Oat*^{rhg} mice (black circles, $n = 6$). Data are shown as means ± SEM. Two-way ANOVA, $^{*}P < 0.05$; $^{***}P < 0.001$. *Abbreviation: ns: not statistically significant difference.*

 © 2023 The Authors

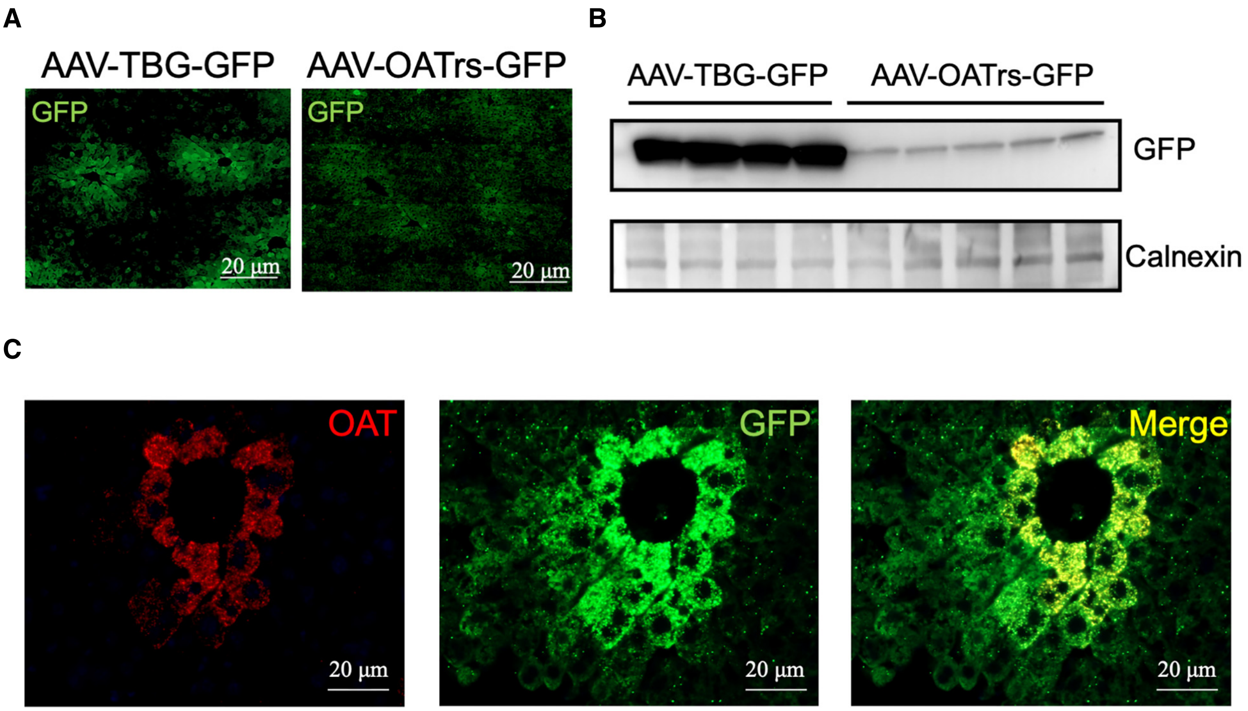

Figure EV2. Patterns of liver transduction.

A Liver section from wild-type C57BL/6N mice systemically injected with an AAV8 vector driving GFP expression under the control of the TBG promoter (AAV-TBG-GFP) or under the control of the OAT regulatory sequence (AAV-OATrs-GFP). Representative images from experimental groups including at least $n = 4$ mice per group are shown. Scale bar: 20 µm.

B Western Blot analysis of lysates from livers of wild-type C57BL/6N mice injected with AAV-TBG-GFP ($n = 4$) or AAV-OATrs-GFP ($n = 5$) vectors.

C Representative immunofluorescence images of livers from wild-type C57BL/6N mice ($n = 4$) injected with AAV-TBG-GFP. OAT staining is show in red and GFP in green, colocalization of the signal is shown in yellow. Scale bar: 20 µm.

Figure EV3. OAT gene transfer in *Oat*^{rhg} pigmented mice injected at 6 weeks of age improves retinal phenotype.

A Plasma ornithine concentrations in *Oat*^{rhg} mice injected with AAV-OAT (red circles, $n = 5$) or AAV-GFP (black circles, $n = 3$) vectors. Averages ± SEM are shown; the two-way ANOVA test was used to perform a statistical comparison between groups; $^{**}P < 0.01$; $^{***}P < 0.001$, $^{****}P < 0.0001$.

B Body weights of *Oat*^{rhg} mice injected at 6 weeks of age; the arrow indicates the time of the injections with AAV-OAT (red circles, $n = 5$) or AAV-GFP (black circles, $n = 3$) vectors; data are shown as means ± SEM; Two-way ANOVA test was used for groups comparison.

C a- and b-waves amplitudes recorded in scotopic conditions plotted as a function of light intensity (log cd * s/m^2) in eyes of 11-month-old *Oat*^{rhg} mice injected with AAV-OAT ($n = 9$, red circles) or AAV-GFP ($n = 4$, black circles) vectors. Wild-type (WT) mice were used as controls ($n = 10$, blue circles). All data are shown as averages ± SEM. Two-way ANOVA test was used for comparison between groups. $^{**}P < 0.01$; $^{***}P < 0.001$, $^{****}P < 0.0001$.

D Plasma ornithine and lysine concentrations of *Oat*^{rhg} mice at 12 months post-injection measured by HPLC. One-way ANOVA test with Tukey correction was used for comparison between groups; data are shown as averages ± SEM. $^{*}P < 0.05$; $^{**}P < 0.01$; $^{****}P < 0.0001$.

E Ornithine concentrations in the eyes of mice injected with AAV-OAT compared with eyes of either wild-type or AAV-GFP controls. Data are shown as z-scores. Means ± SEM are shown. One-way ANOVA with False Discovery Rate correction was used for comparison between groups. $^{**}P < 0.01$, $^{****}P < 0.0001$.

F Western blotting for OAT and GFP on livers of *Oat*^{rhg} mice harvested 12 months after the injections of AAV-OAT or AAV-GFP vectors. A WT mouse was included as control, and Vinculin was used as loading control.

G Morphological analysis of *Oat*^{rhg} mice retinas 12 months post-injection of AAV-GFP or AAV-OAT. An age-matched WT mouse is shown as control (left panel). Semi-thin sections (40×). Abbreviations: ns, not statistically significant difference; RPE, retinal pigment epithelium; OS, outer segment; IS, inner segment; ONL, outer nuclear layer; WT, wild-type.

© 2023 The Authors

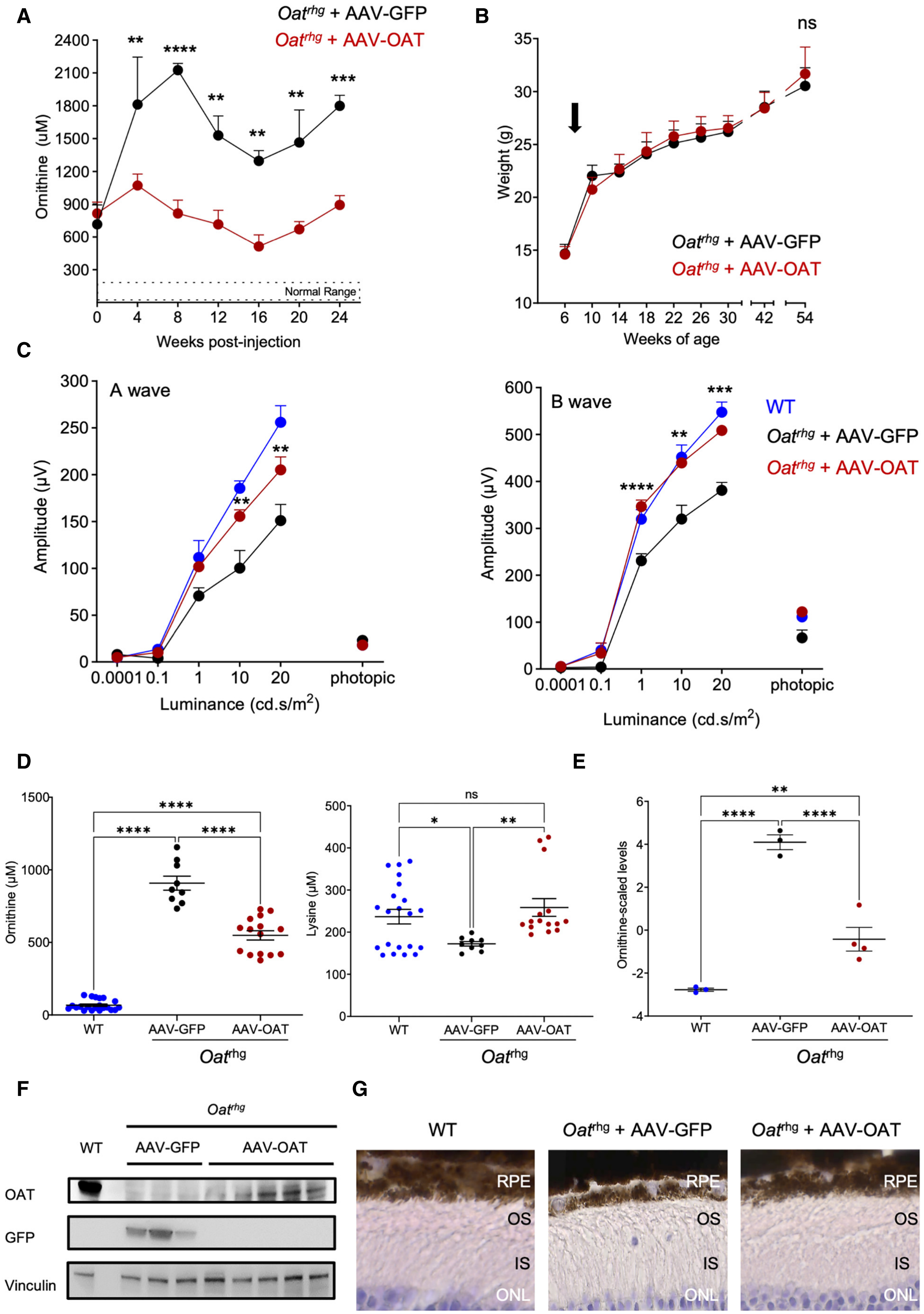

Figure EV3.

© 2023 The Authors

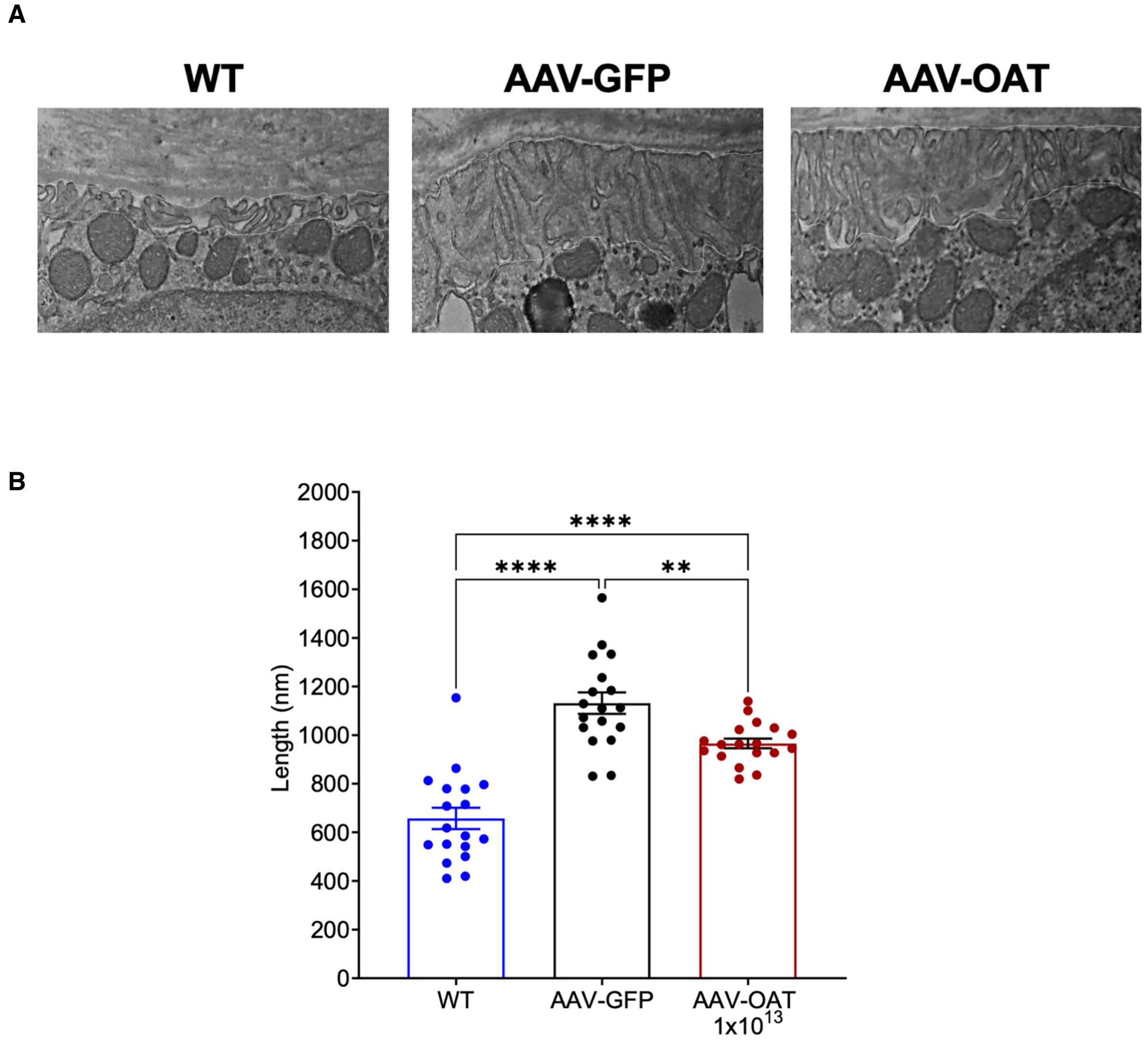

Figure EV4. Electron microscopy of basal infoldings of Bruch's membrane of *Oat^{rhg}* mice.

A AAV-GFP eyes (middle panel) displaying irregular and highly altered thickened ultrastructure of basal infoldings compared with wild-type (WT-left panel) or AAV-OAT (dose is shown as gc/kg) injected mice (right panel). Basal infoldings of Bruch's membrane is outlined by dashed line. Scale bar: 750 nm.

B Quantification of basal membrane thickness. Layers' depth was measured and averaged from six fields for each group, three measurements for each image. One mouse was analyzed for each group. One-way ANOVA test with Tukey correction was performed to compare experimental groups. Means ± SEM are shown **$P < 0.01$; ****$P < 0.0001$.

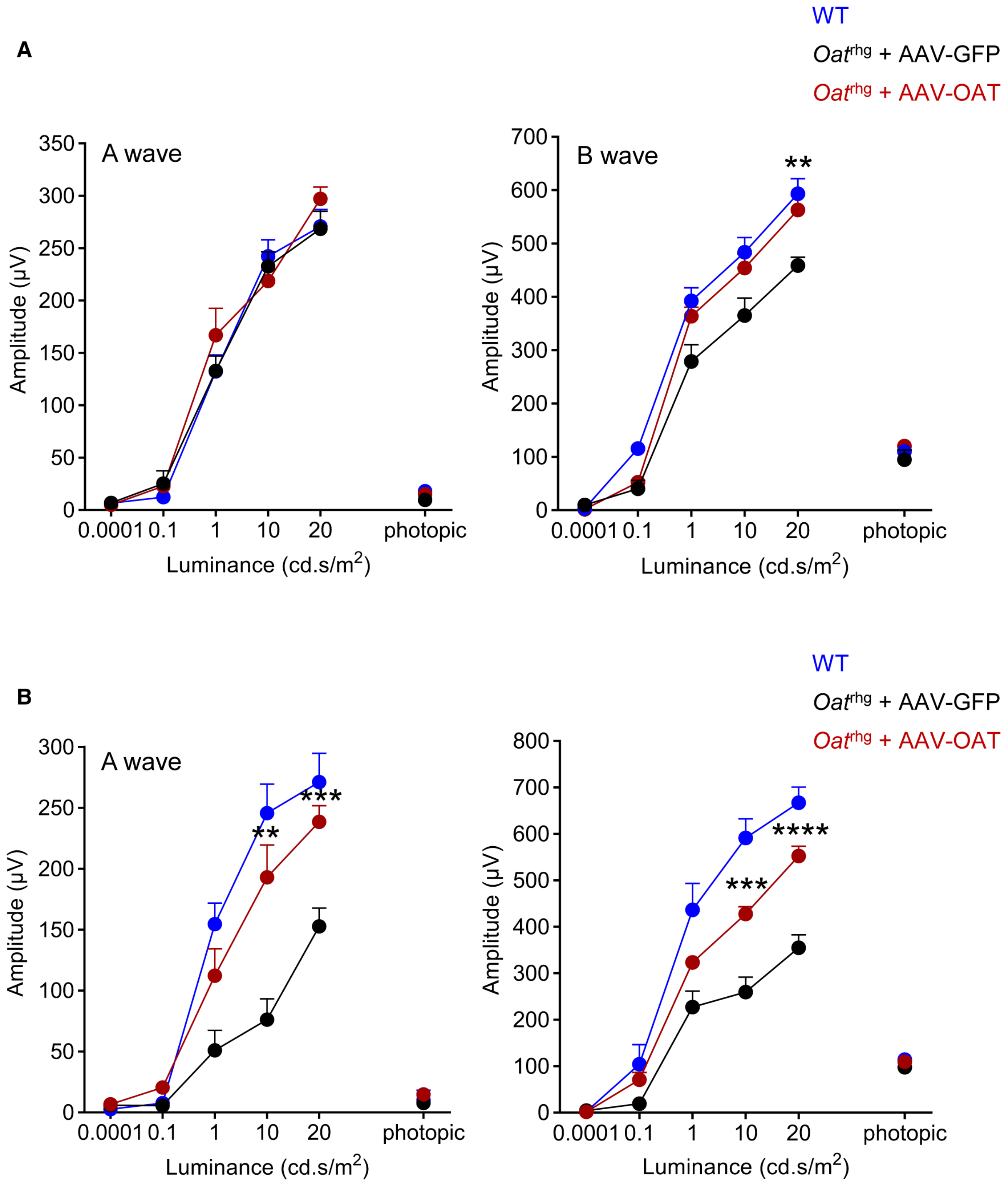

Figure EV5. AAV-mediated liver-directed OAT delivery into *Oat*^{rhg} albino mice.

A, B ERG components (a- and b- waves) under scotopic and photopic conditions of 7-month-old (A) and 11-month-old (B) *Oat*^{rhg} albino mice injected with 1 x 10^{13} gc/kg of AAV-OAT (n = 10, 7-month-old; n = 7, 11-month-old) or AAV-GFP vectors (n = 6, 7-month-old; n = 7, 11-month-old). Wild-type (WT) mice were included as control (n = 7, 7-month-old; n = 3, 11-month-old). All data are shown as averages ± SEM. Two-way ANOVA test was used for comparison between groups. **$P < 0.01$; ***$P < 0.001$, ****$P < 0.0001$.

 © 2023 The Authors
