# Supplementary material for: Liver‐directed gene therapy for ornithine aminotransferase deficiency
Source: EMBO Mol Med. 2023 Jan 17;15(4):e17033. doi: 10.15252/emmm.202217033 (PMC10086579; doi:10.15252/emmm.202217033)
Supplement: Supplementary file 2 — Table EV1 [file EMMM-15-e17033-s003.docx]

| Plasma A.A. (µM) | WT | | AAV-OAT | | AAV-GFP | |
| --- | --- | --- | --- | --- | --- | --- |
|  | Average | SD | Average | SD | Average | SD |
| ASP | 11.5 | 8.2 | 8.5 | 2.9 | 6.8 | 2.2 |
| GLU | 68.0 | 24.3 | 72.0 | 12.5 | 65.0 | 8.1 |
| ASN | 59.9 | 22.7 | 49.6 | 16.8 | 43.1^§^ | 4.3 |
| SER | 100.4 | 18.6 | 135.5^§^ | 35.1 | 117.5^§^ | 9.2 |
| GLN | 680.9 | 117.9 | 733.8 | 71.7 | 657.9^¥^ | 55.6 |
| HIS | 71.8 | 13.3 | 89.98^§^ | 6.2 | 75.7^¥^ | 10.3 |
| GLY | 191.9 | 25.9 | 253.2^§^ | 39.3 | 227.5^§^ | 29.7 |
| THR | 120.4 | 29.3 | 146.4 | 37.9 | 139.5 | 22.5 |
| CIT | 61.1 | 17.7 | 73.1 | 13.6 | 74.4 | 26.0 |
| ARG | 64.5 | 30.2 | 119.9^§^ | 22.9 | 128.8^§^ | 22.6 |
| ALA | 368.5 | 63.1 | 499.2^§^ | 142.9 | 448.3^§^ | 31.4 |
| TYR | 68.1 | 19.7 | 74.9 | 14.7 | 80.9 | 9.4 |
| VAL | 198.1 | 42.5 | 182.1 | 24.3 | 221.1^¥^ | 22.6 |
| MET | 50.2 | 16.8 | 56.1 | 19.1 | 71.6^§^ | 11.5 |
| TRP | 31.5 | 9.7 | 21.2^§^ | 10.6 | 25.5^§^ | 3.2 |
| PHE | 82.7 | 14.3 | 79.3 | 13.8 | 87.5 | 9.3 |
| ILE | 87.3 | 18.6 | 83.6 | 15.0 | 99.5^§, ¥^ | 5.8 |
| LEU | 134.6 | 34.4 | 126.9 | 21.6 | 149.6 | 8.4 |
| PRO | 99.0 | 25.9 | 109.3 | 45.3 | 118.0^§, ¥^ | 9.1 |

**Table EV1.** Plasma amino acids (µM) in *Oat^rhg^* mice injected with 1x10^13^ gc/kg AAV-OAT or AAV-GFP at 12-month post-injection. Wild-type (WT) control mice are also shown. ^§^Mean value significantly different from WT (p<0.05). ^¥^AAV-GFP mean value significantly different from AAV-OAT (p<0.05).
