# Supplementary figures and images for: Liver‐directed gene therapy for ornithine aminotransferase deficiency
Source: EMBO Mol Med. 2023 Jan 17;15(4):e17033. doi: 10.15252/emmm.202217033 (PMC10086579; doi:10.15252/emmm.202217033)

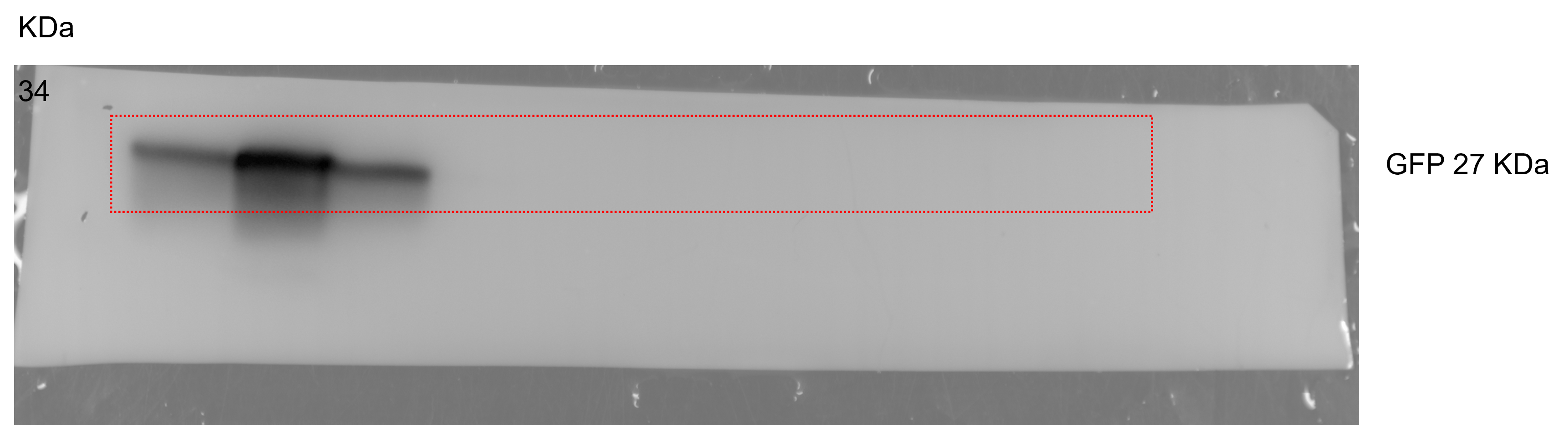

Supplement: Supplementary file 4 — Source Data for Figure 1 [file EMMM-15-e17033-s001.zip › Figure 1/1E/GFP.tif]

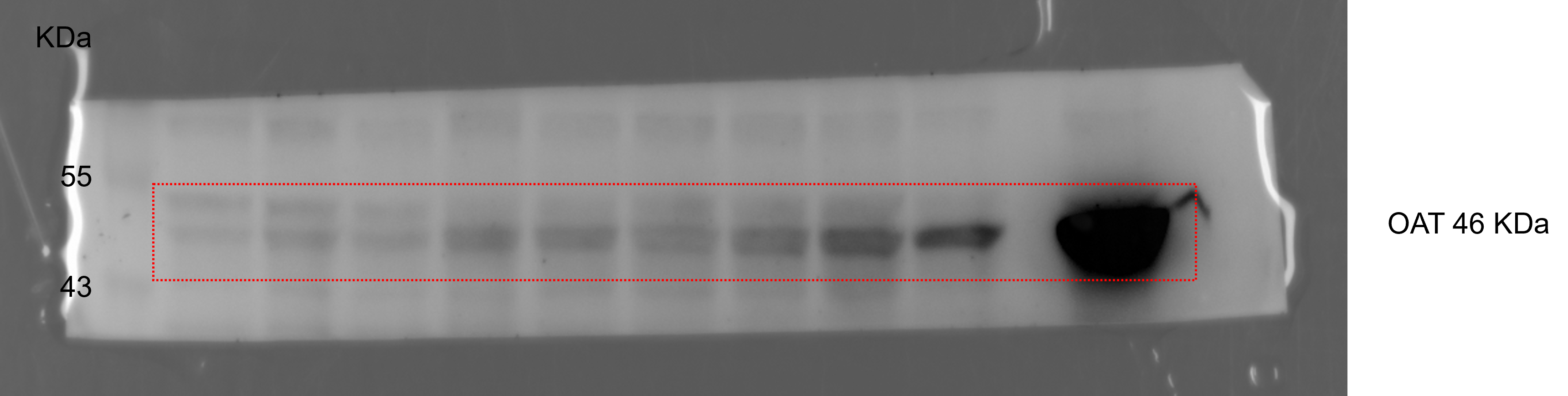

Supplement: Supplementary file 4 — Source Data for Figure 1 [file EMMM-15-e17033-s001.zip › Figure 1/1E/OAT.tif]

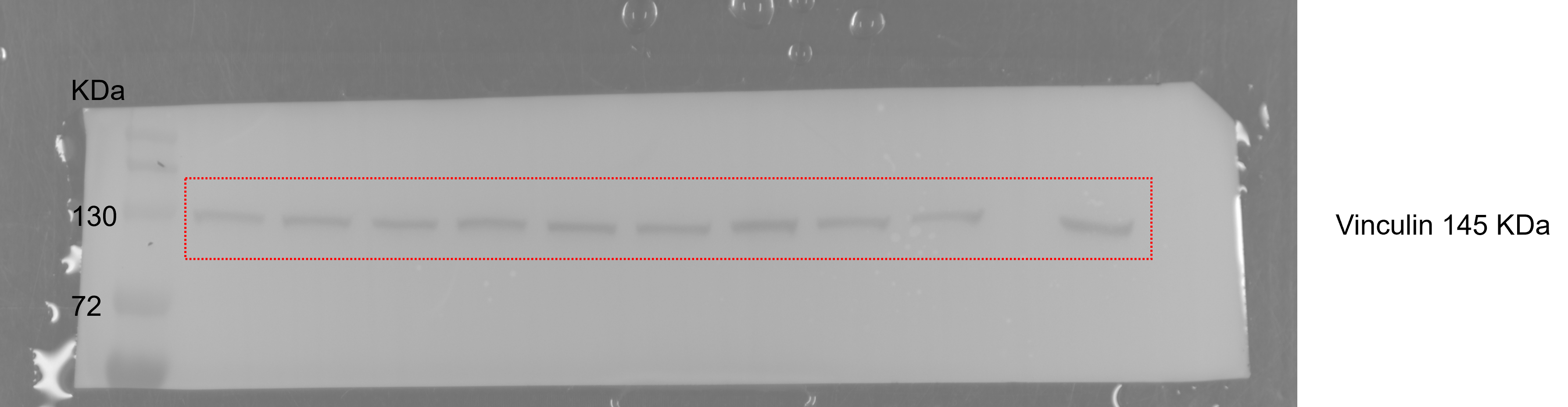

Supplement: Supplementary file 4 — Source Data for Figure 1 [file EMMM-15-e17033-s001.zip › Figure 1/1E/Vinculin.tif]

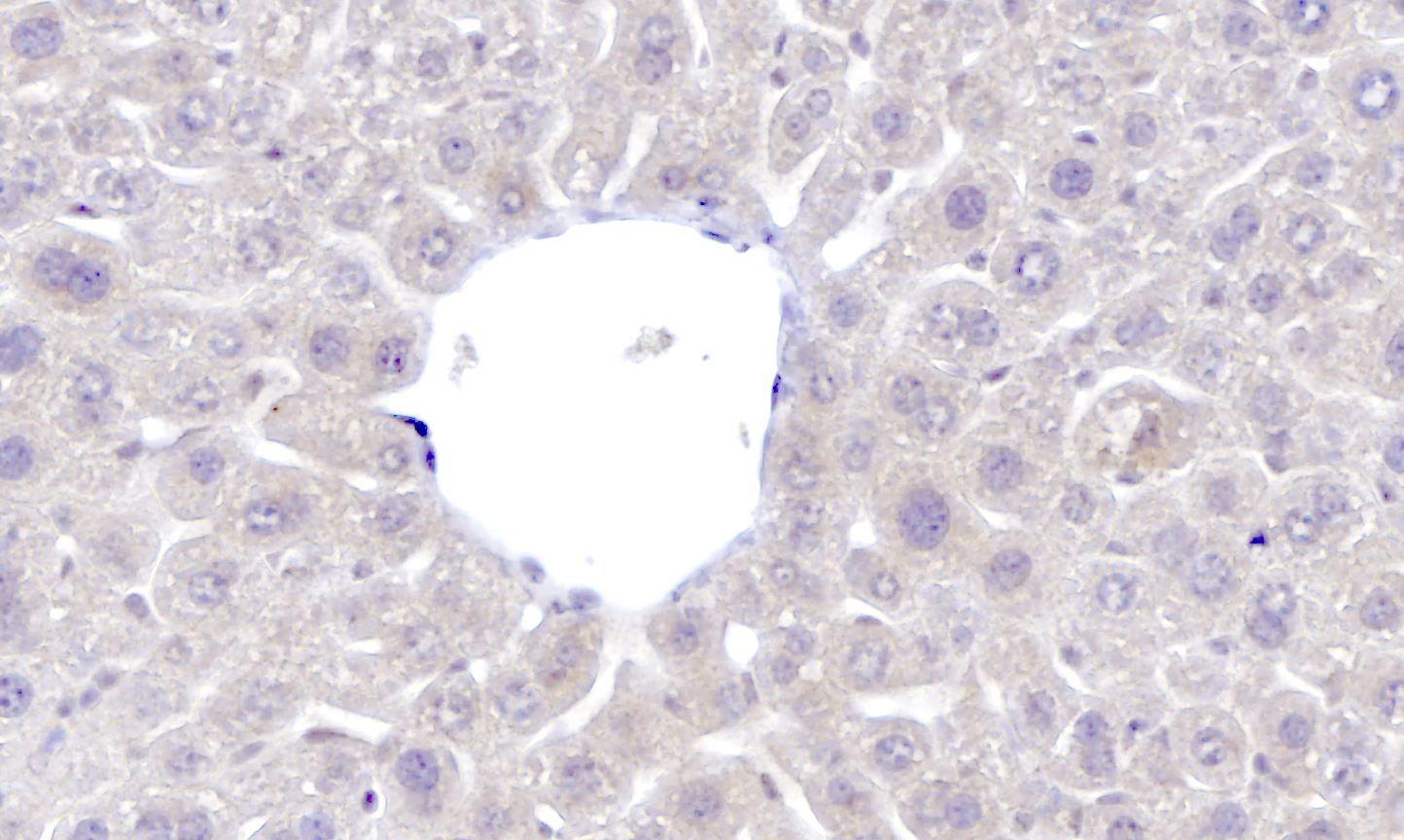

Supplement: Supplementary file 4 — Source Data for Figure 1 [file EMMM-15-e17033-s001.zip › Figure 1/1G/AAV-GFP 20X.tiff]

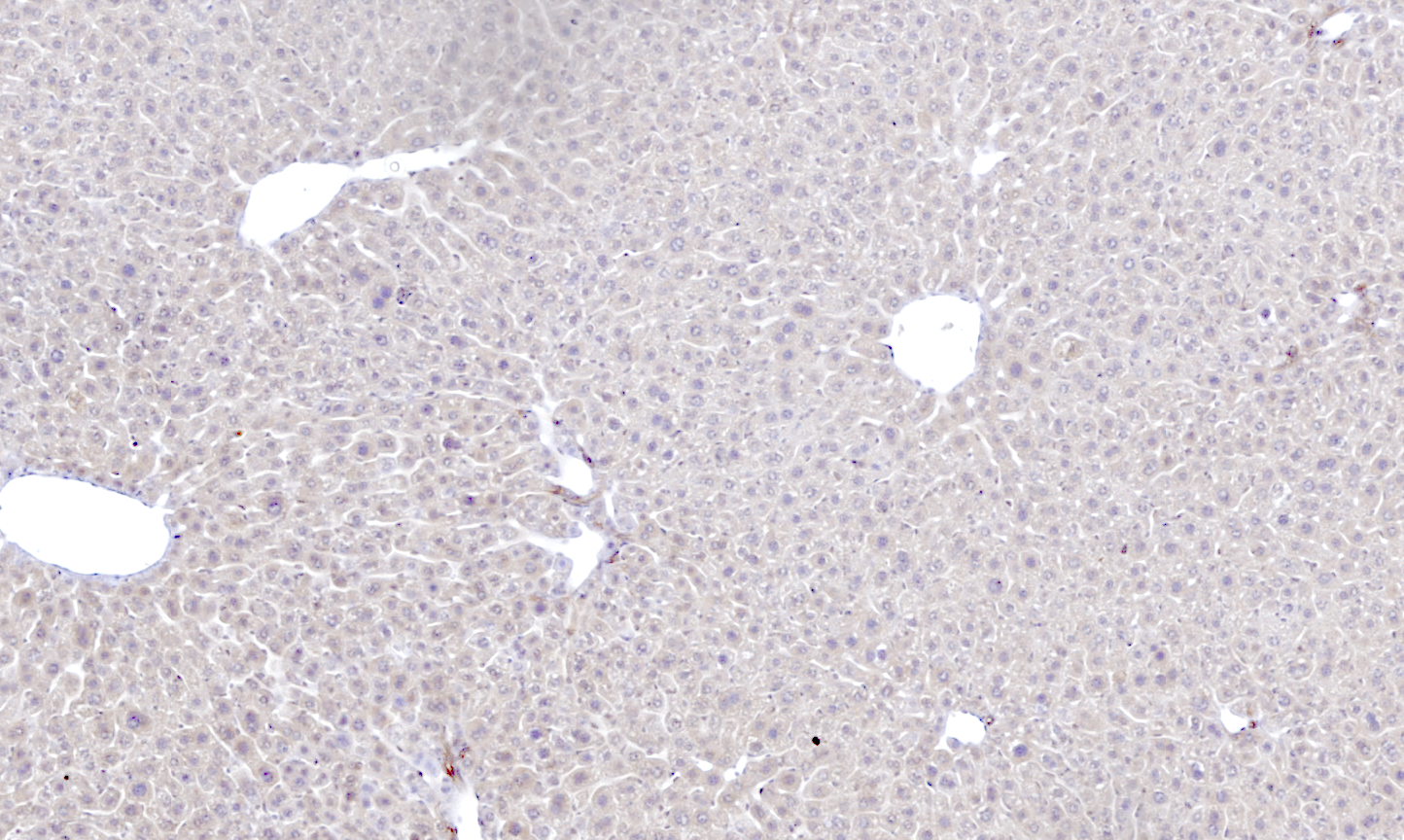

Supplement: Supplementary file 4 — Source Data for Figure 1 [file EMMM-15-e17033-s001.zip › Figure 1/1G/AAV-GFP 5X.tiff]

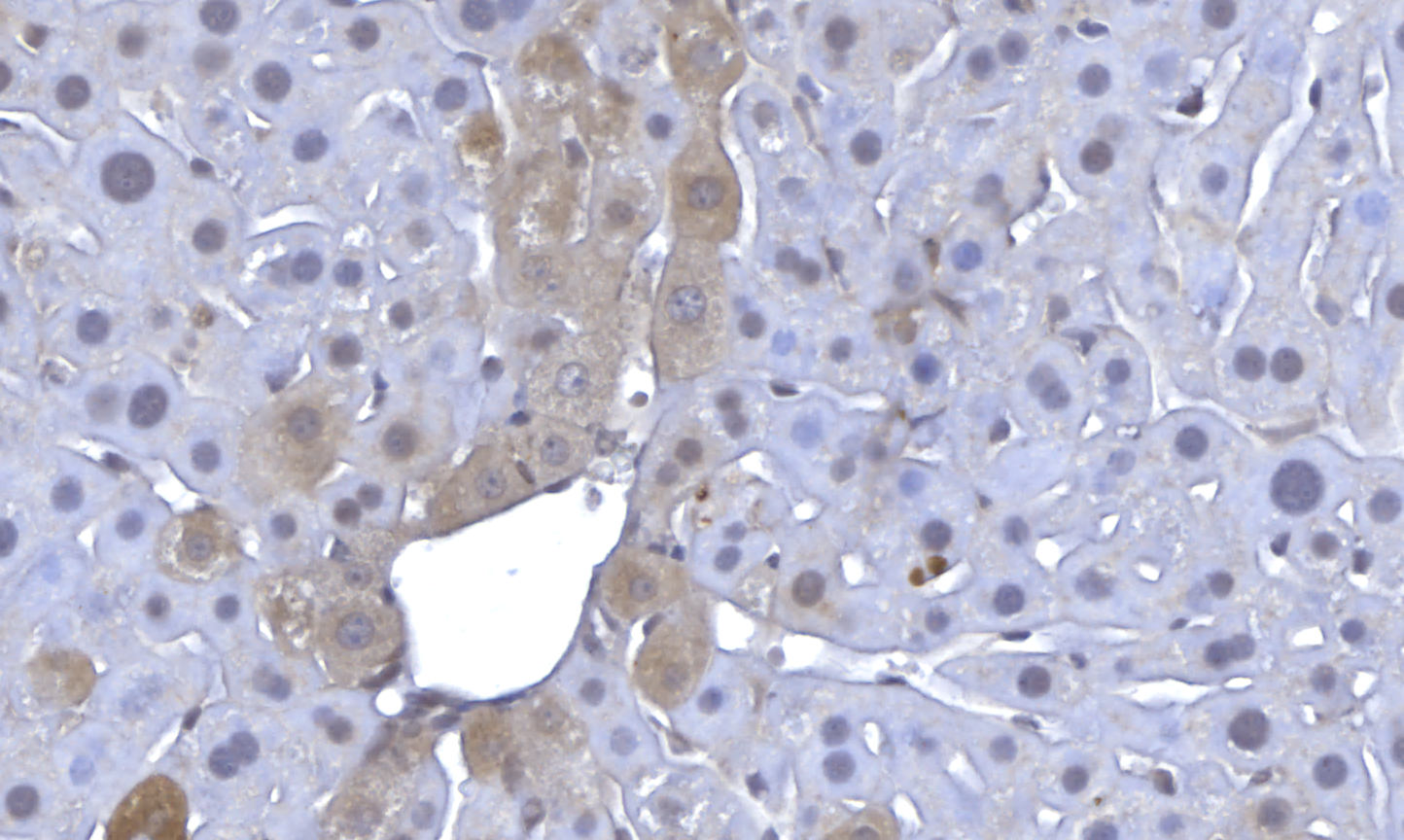

Supplement: Supplementary file 4 — Source Data for Figure 1 [file EMMM-15-e17033-s001.zip › Figure 1/1G/AAV-OAT 20X.tiff]

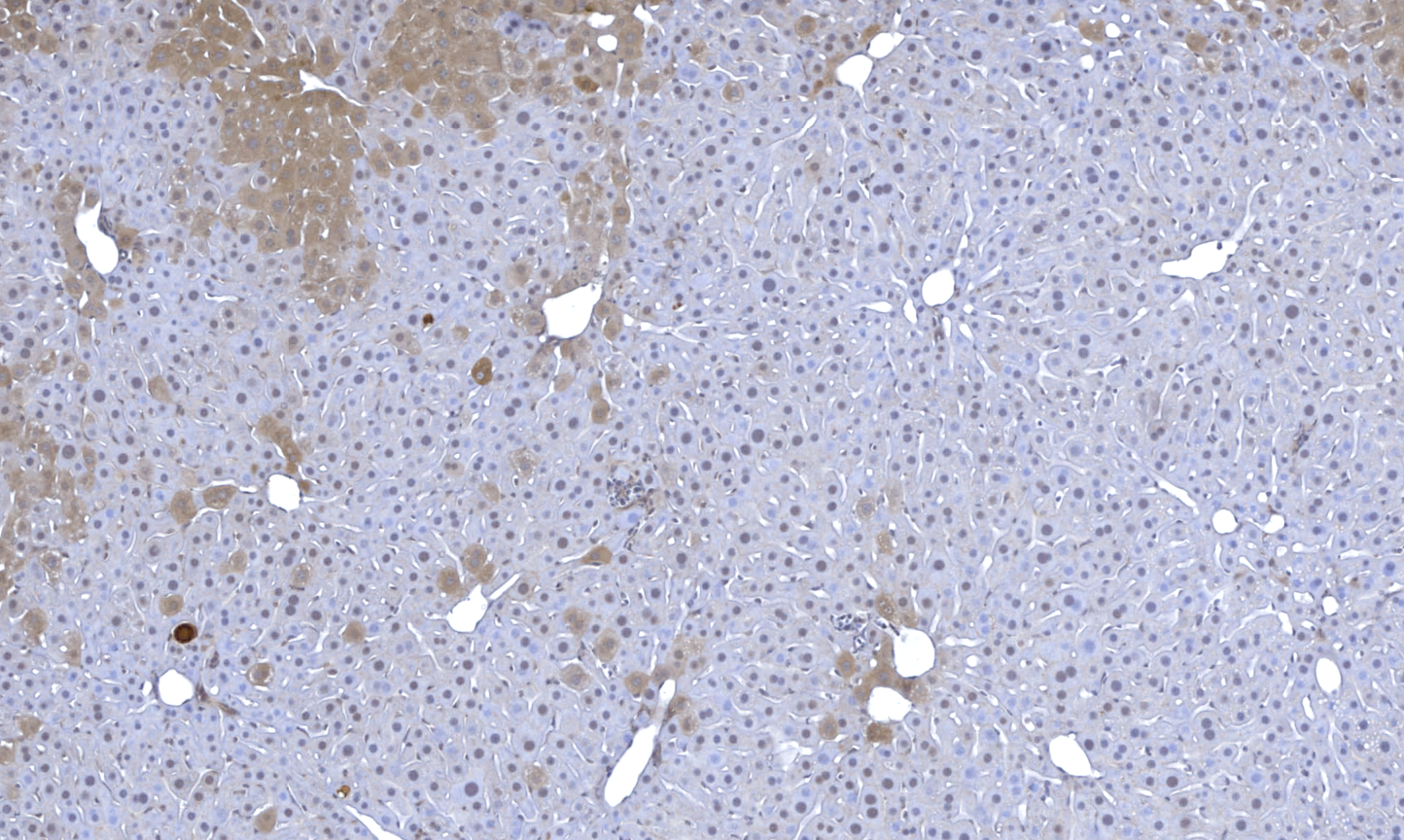

Supplement: Supplementary file 4 — Source Data for Figure 1 [file EMMM-15-e17033-s001.zip › Figure 1/1G/AAV-OAT 5X.tiff]

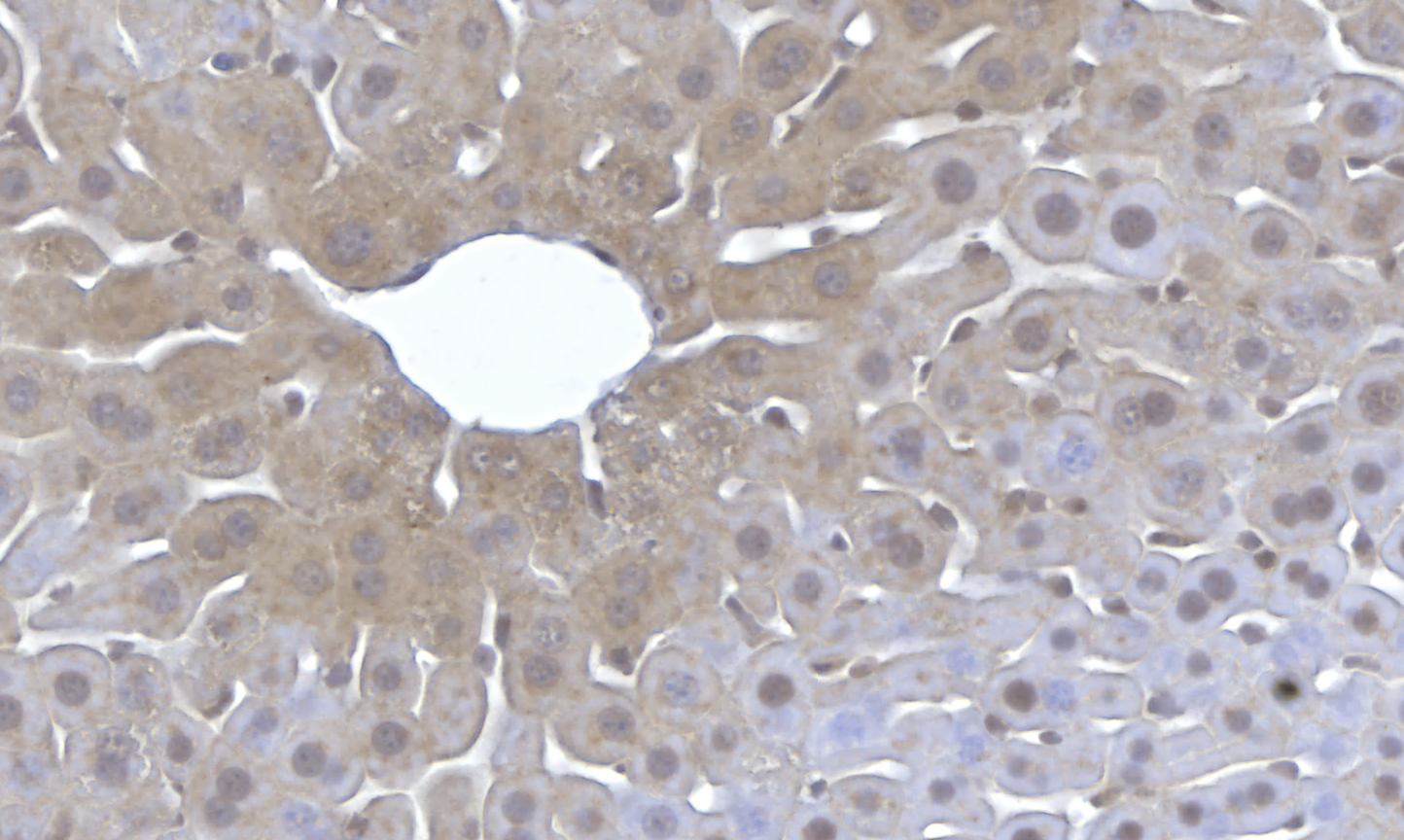

Supplement: Supplementary file 4 — Source Data for Figure 1 [file EMMM-15-e17033-s001.zip › Figure 1/1G/WT 20X.tiff]

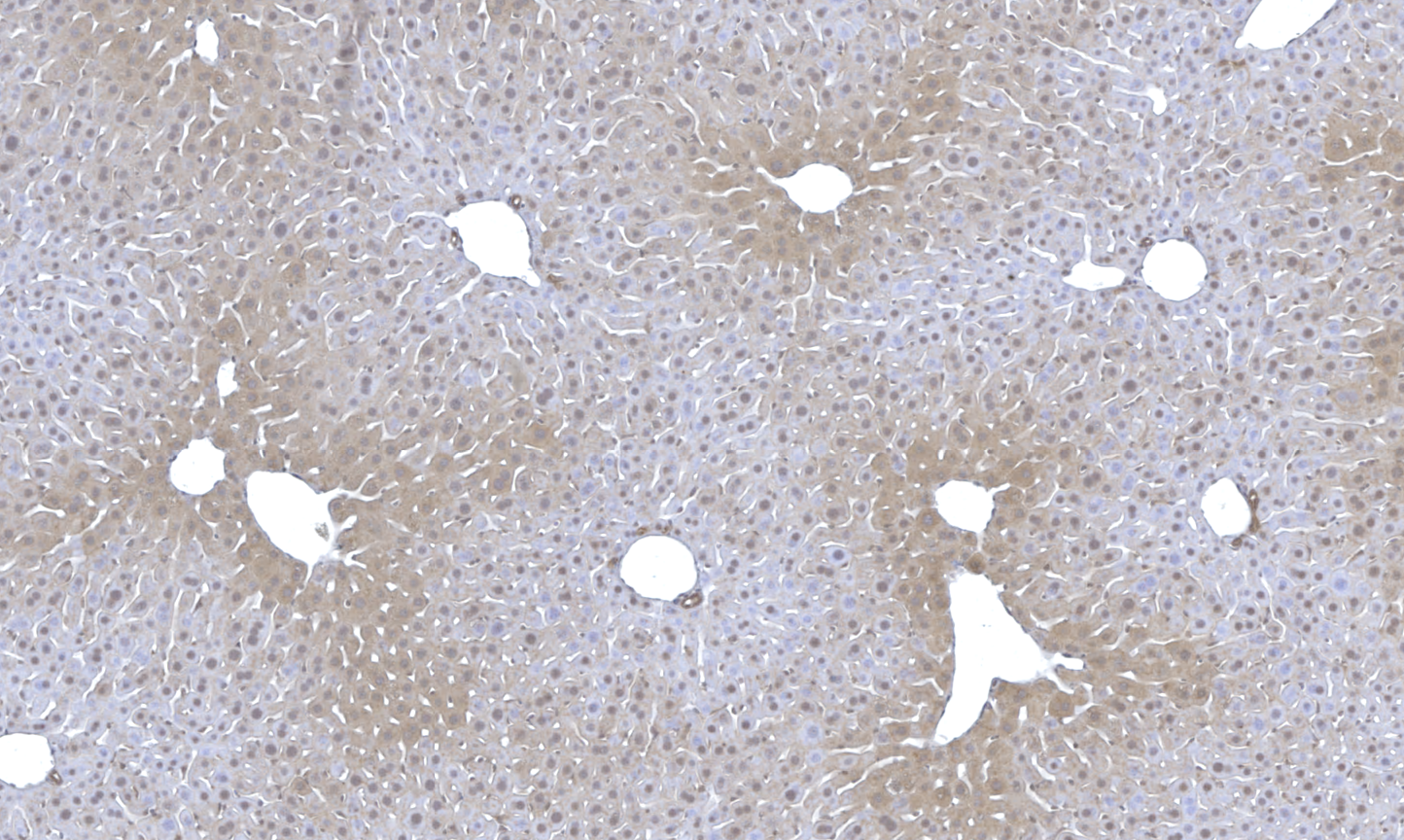

Supplement: Supplementary file 4 — Source Data for Figure 1 [file EMMM-15-e17033-s001.zip › Figure 1/1G/WT 5X.tiff]

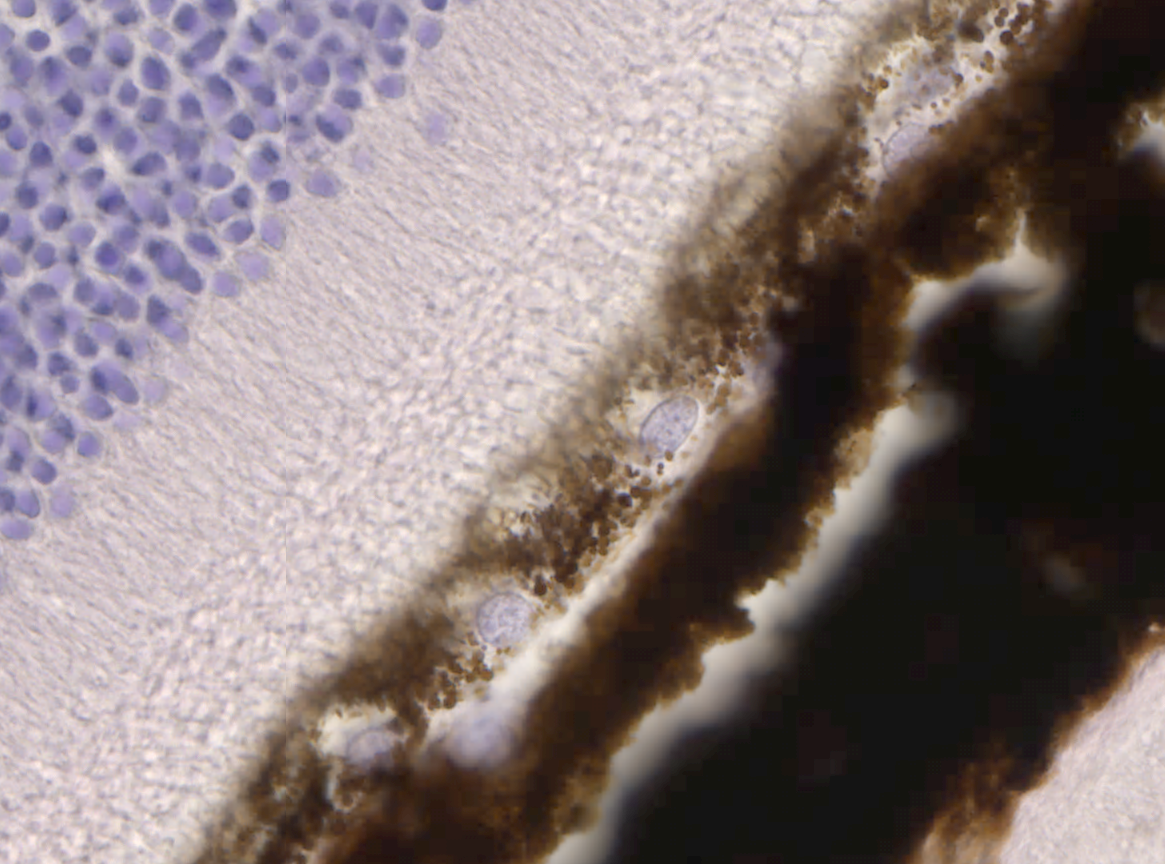

Supplement: Supplementary file 4 — Source Data for Figure 1 [file EMMM-15-e17033-s001.zip › Figure 1/1H/Oat rhg AAV-GFP.tif]

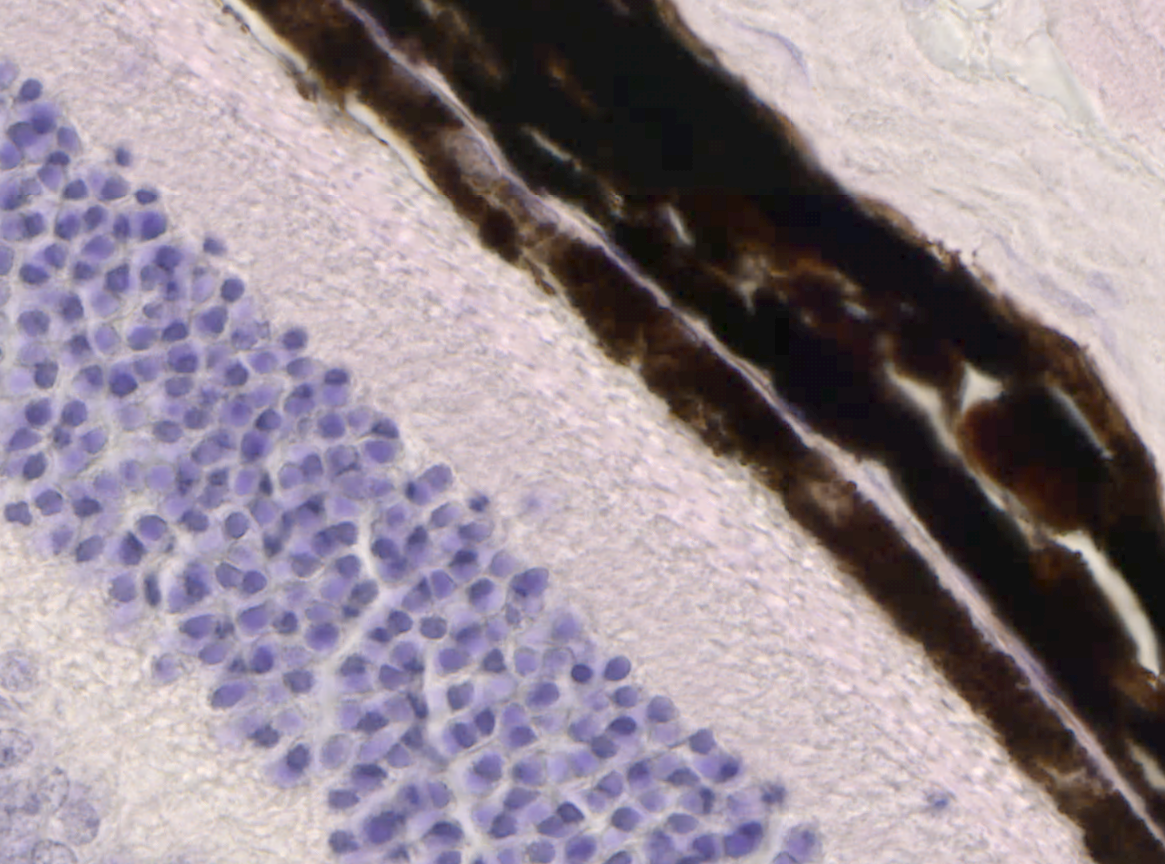

Supplement: Supplementary file 4 — Source Data for Figure 1 [file EMMM-15-e17033-s001.zip › Figure 1/1H/Oat rhg AAV-OAT 1x10e13.tif]

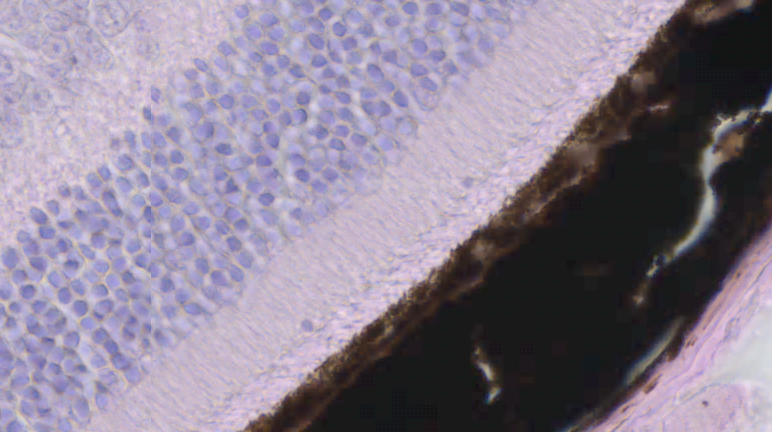

Supplement: Supplementary file 4 — Source Data for Figure 1 [file EMMM-15-e17033-s001.zip › Figure 1/1H/Oat rhg AAV-OAT 3x10e13.tiff]

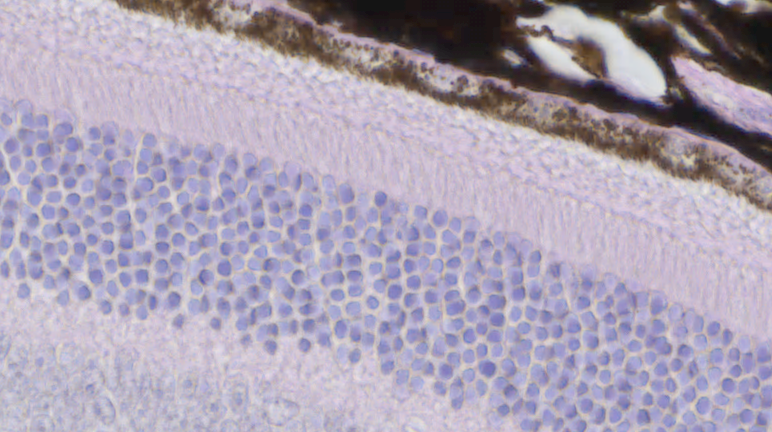

Supplement: Supplementary file 4 — Source Data for Figure 1 [file EMMM-15-e17033-s001.zip › Figure 1/1H/Oatrhg.tiff]

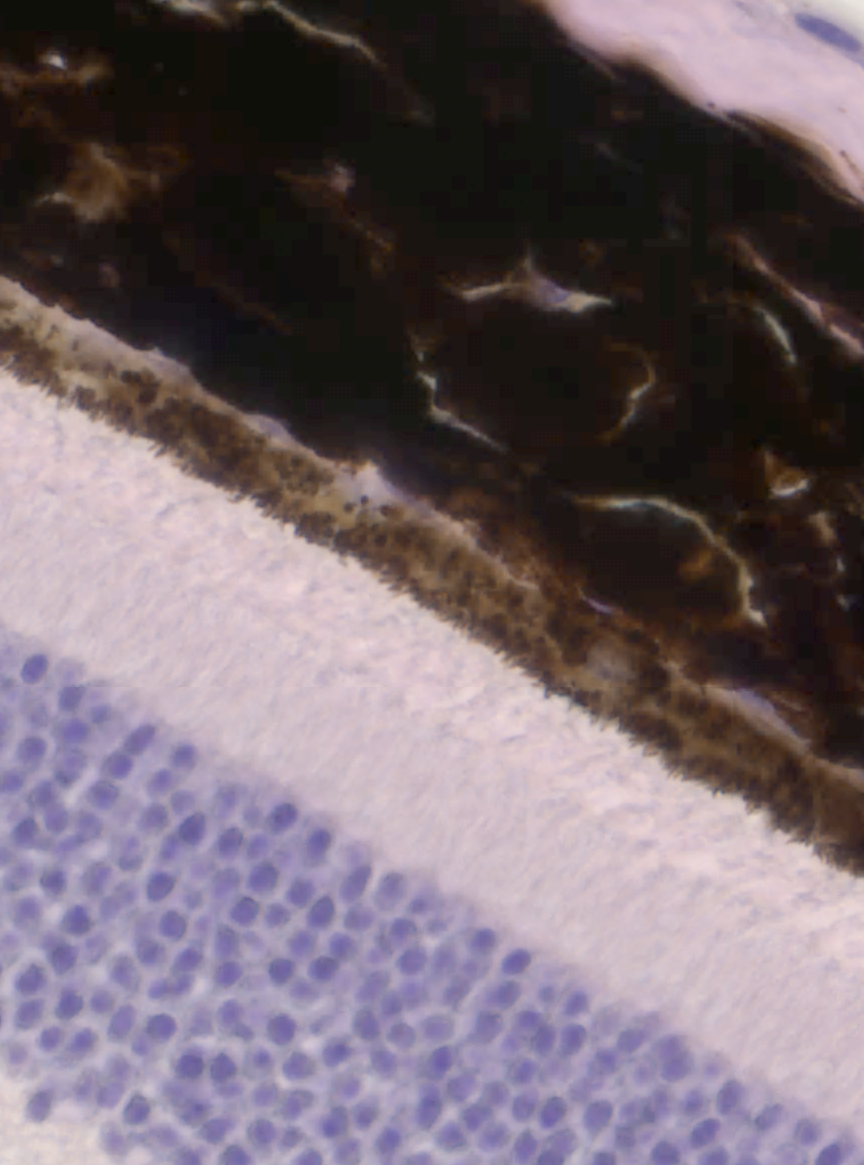

Supplement: Supplementary file 4 — Source Data for Figure 1 [file EMMM-15-e17033-s001.zip › Figure 1/1H/WT.tif]

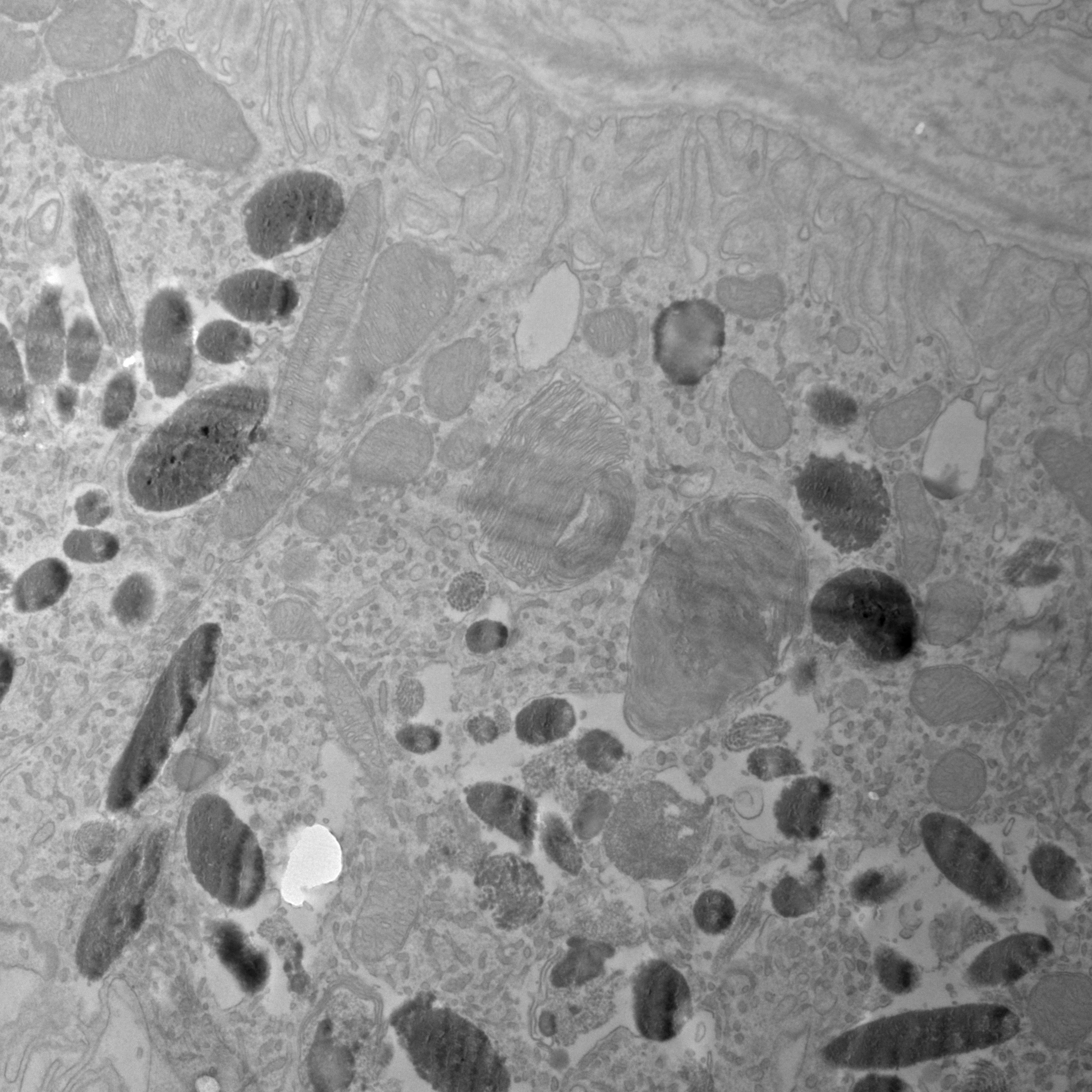

Supplement: Supplementary file 5 — Source Data for Figure 2 [file EMMM-15-e17033-s008.zip › Figure 2/2A/AAV-GFP 1.tif]

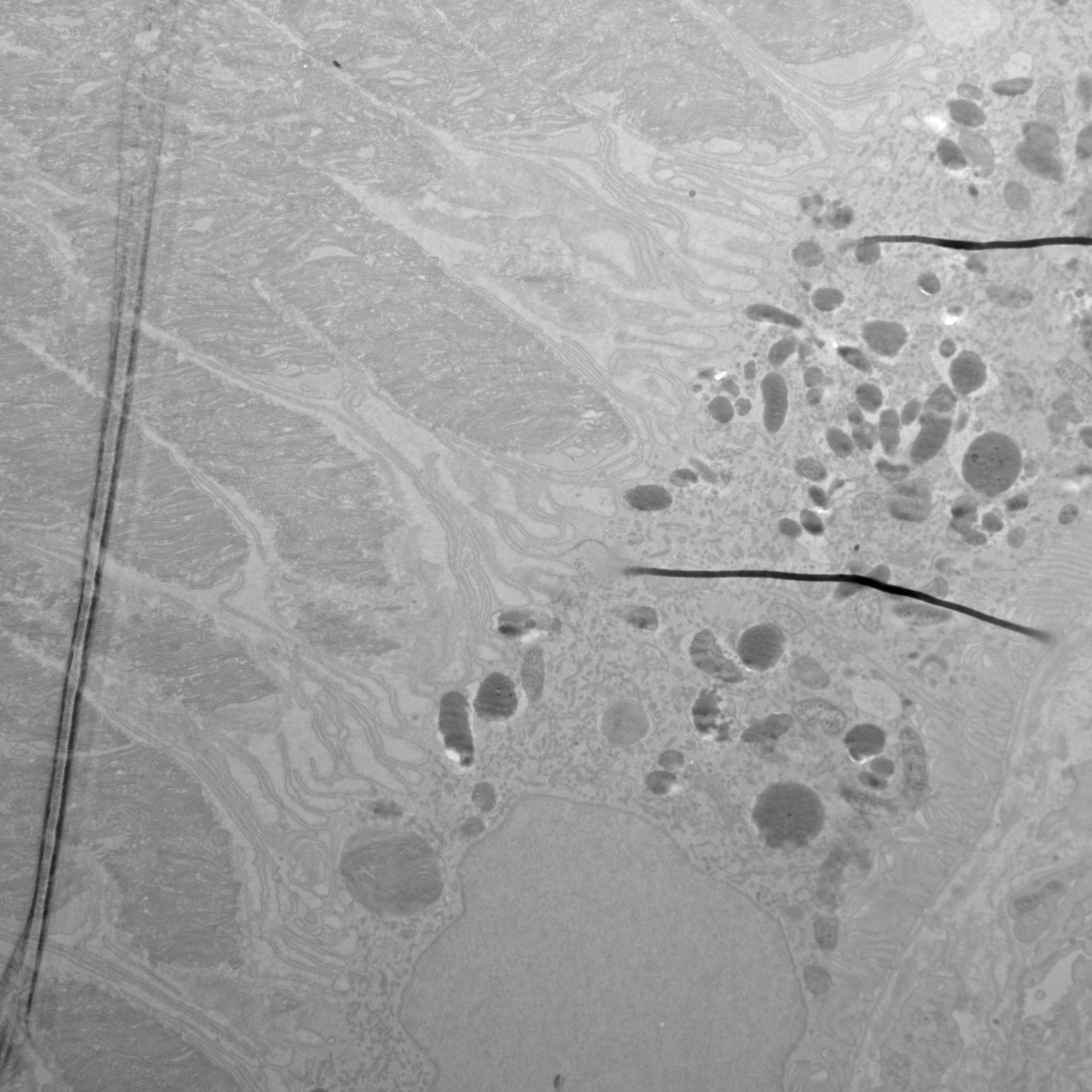

Supplement: Supplementary file 5 — Source Data for Figure 2 [file EMMM-15-e17033-s008.zip › Figure 2/2A/AAV-GFP 2.tif]

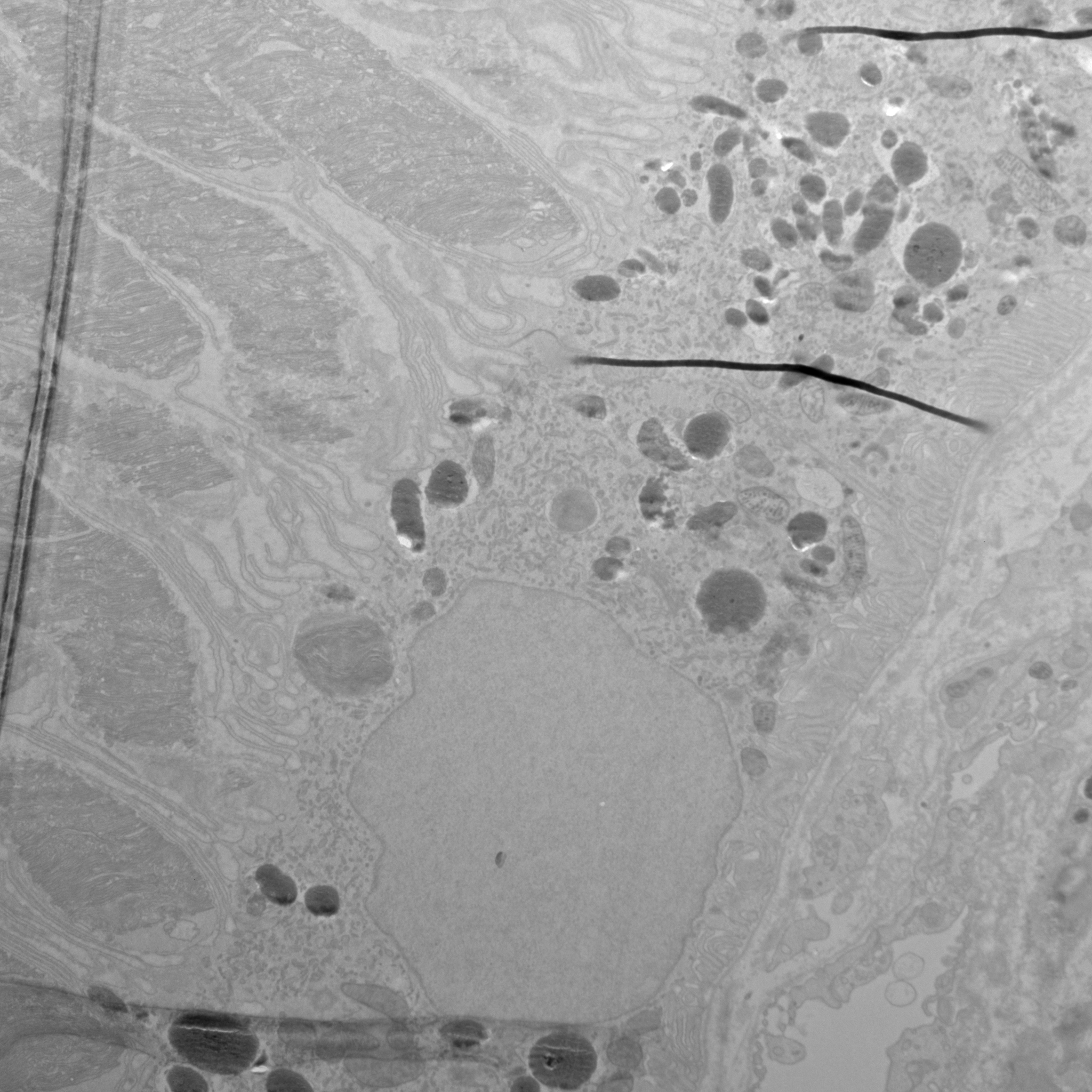

Supplement: Supplementary file 5 — Source Data for Figure 2 [file EMMM-15-e17033-s008.zip › Figure 2/2A/AAV-GFP 3.tif]

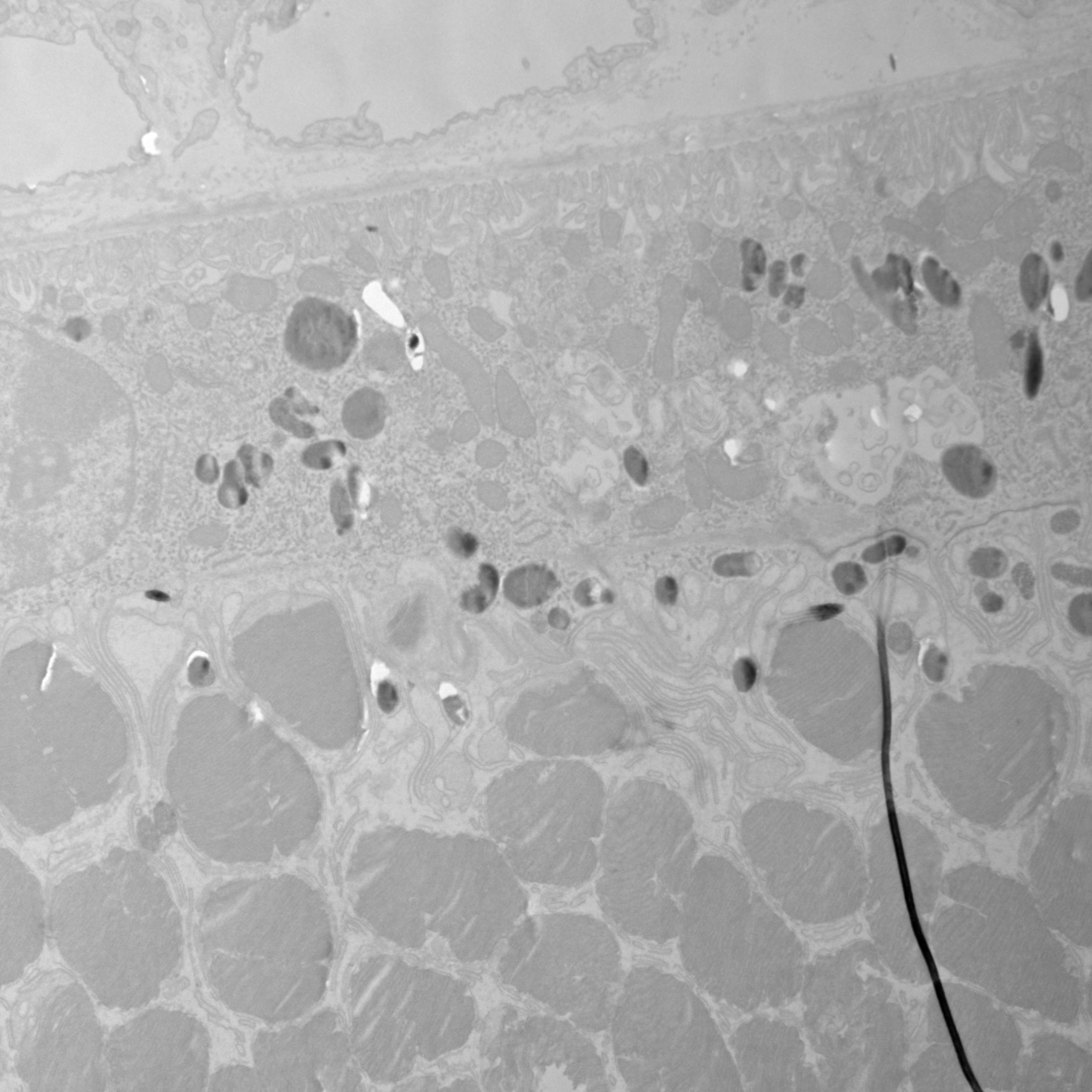

Supplement: Supplementary file 5 — Source Data for Figure 2 [file EMMM-15-e17033-s008.zip › Figure 2/2B/AAV-OAT 1.tif]

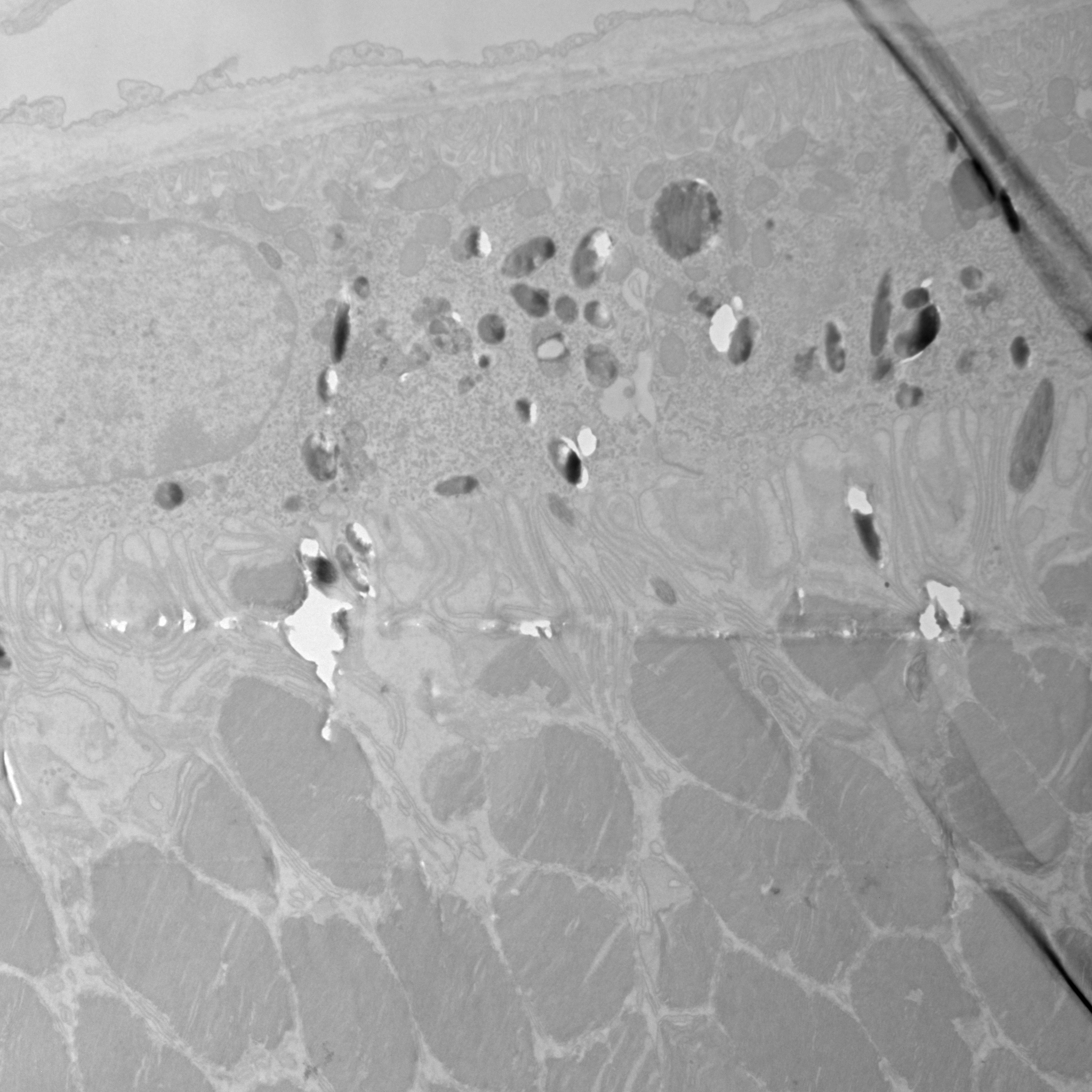

Supplement: Supplementary file 5 — Source Data for Figure 2 [file EMMM-15-e17033-s008.zip › Figure 2/2B/AAV-OAT 2.tif]

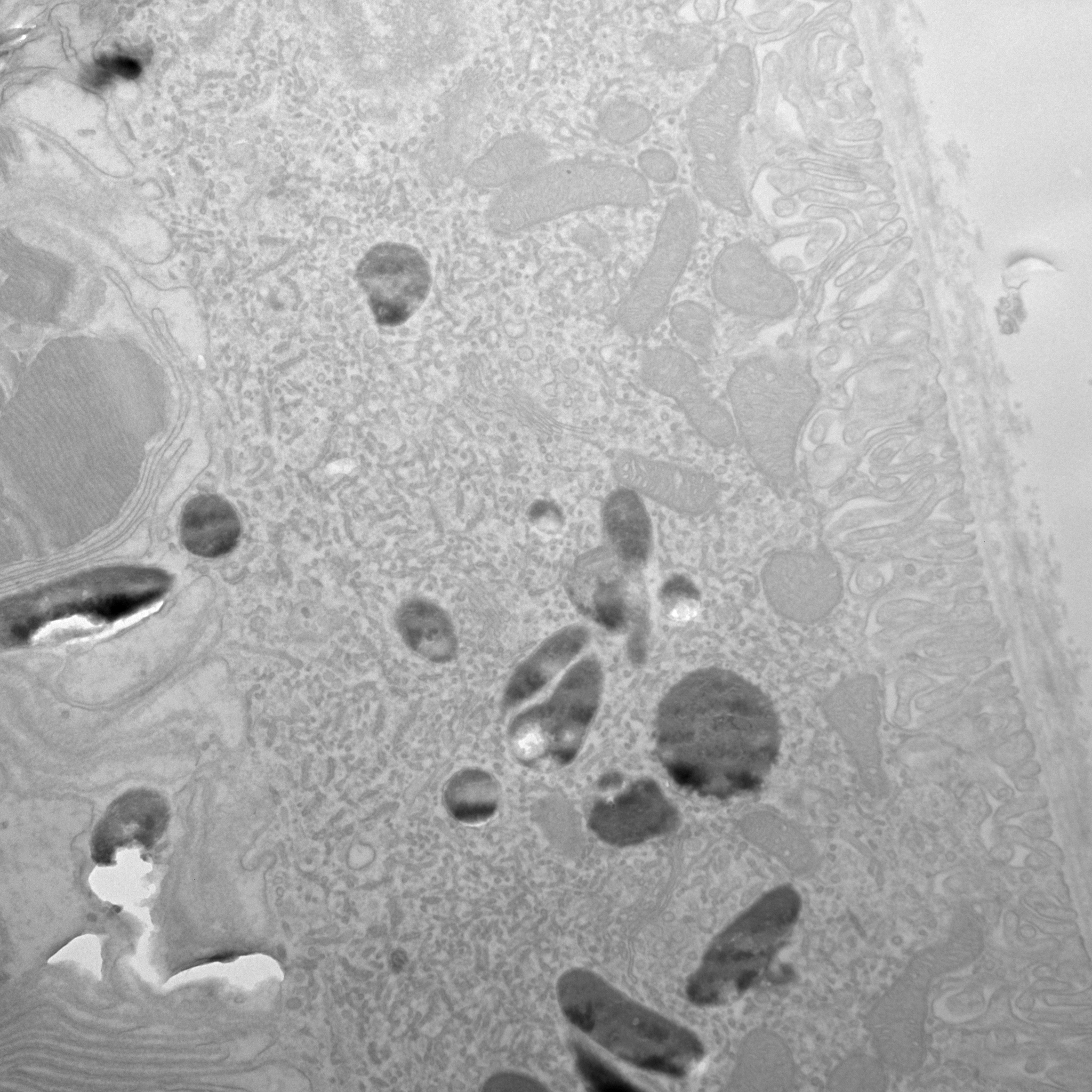

Supplement: Supplementary file 5 — Source Data for Figure 2 [file EMMM-15-e17033-s008.zip › Figure 2/2B/AAV-OAT 3.tif]

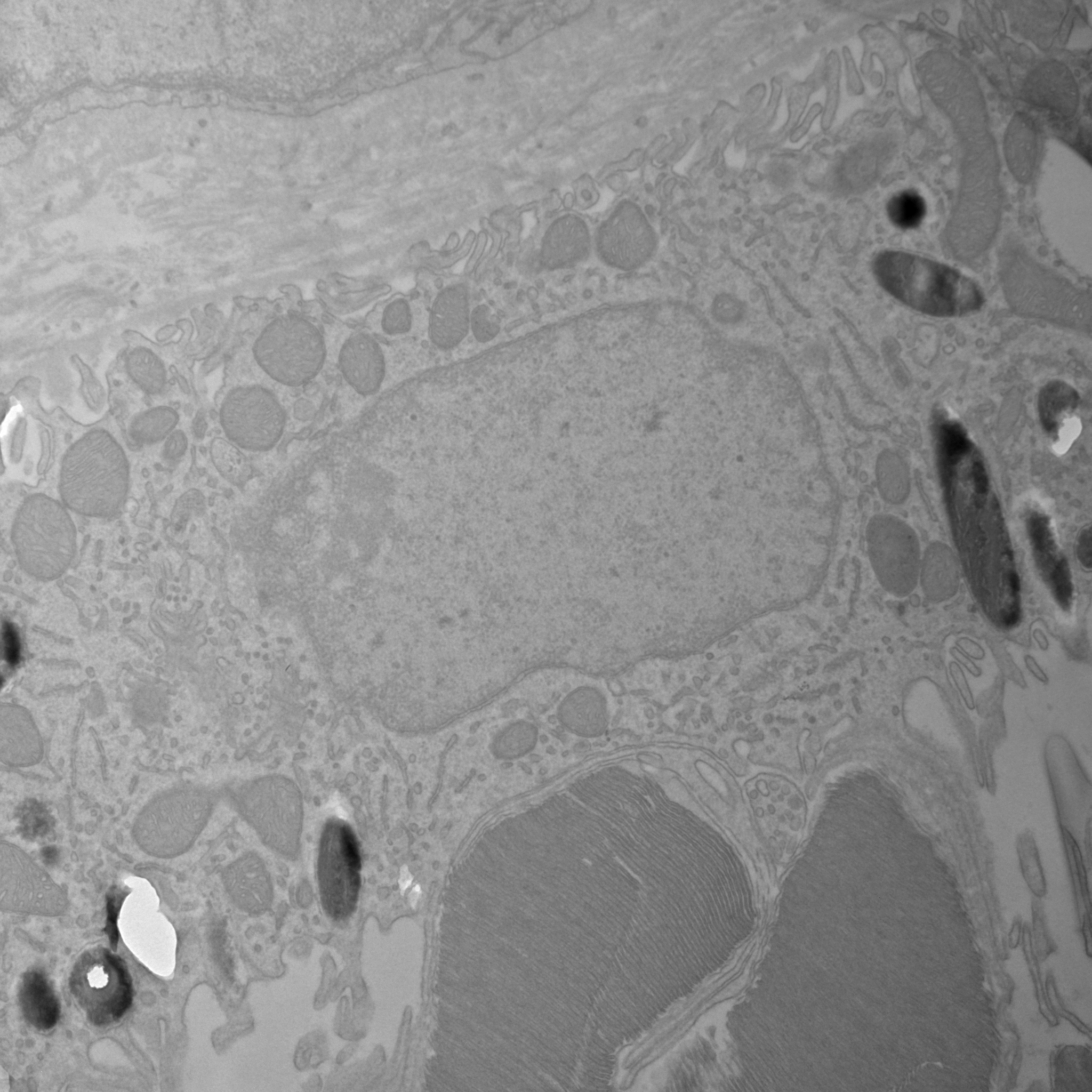

Supplement: Supplementary file 5 — Source Data for Figure 2 [file EMMM-15-e17033-s008.zip › Figure 2/2C/WT 1.tif]

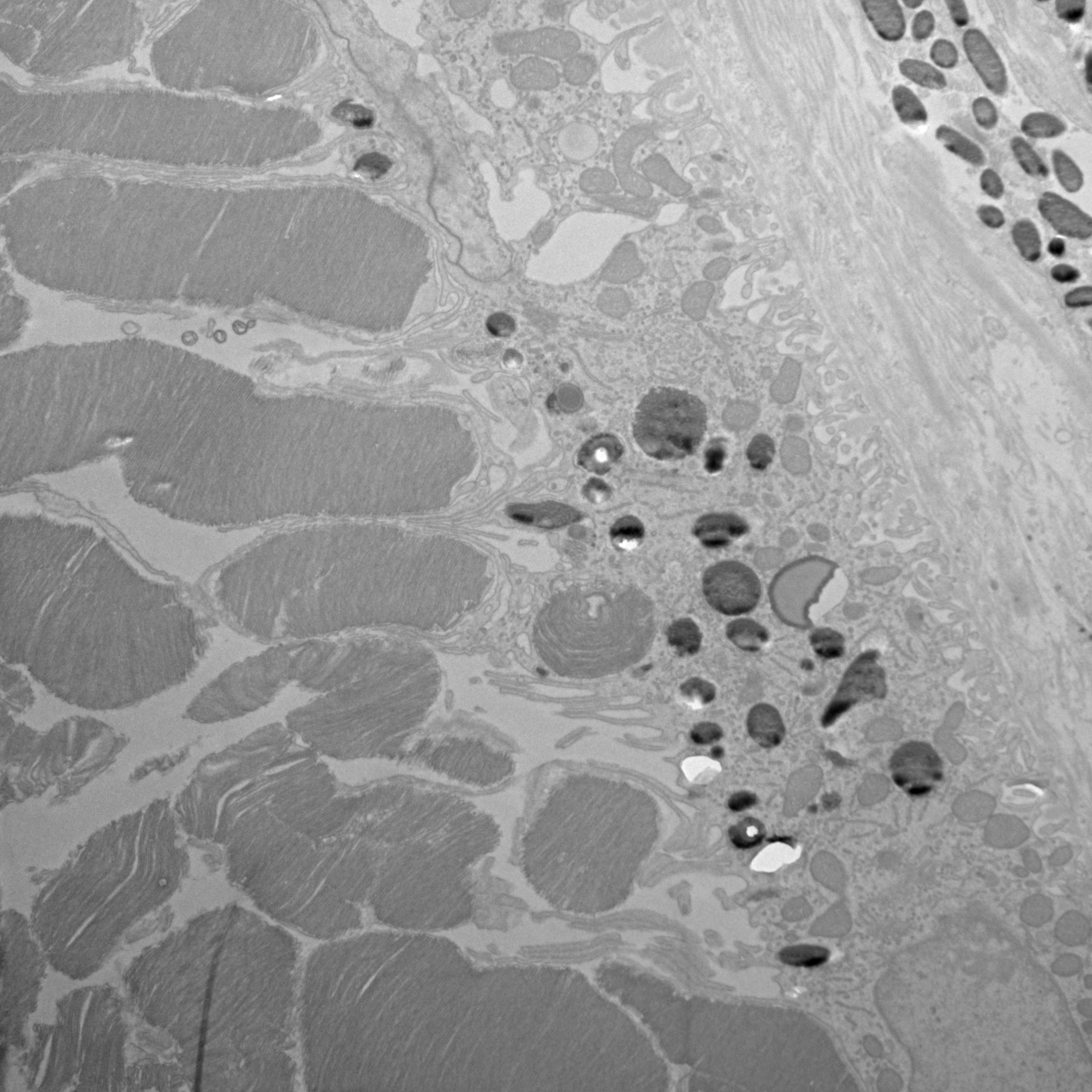

Supplement: Supplementary file 5 — Source Data for Figure 2 [file EMMM-15-e17033-s008.zip › Figure 2/2C/WT 2.tif]

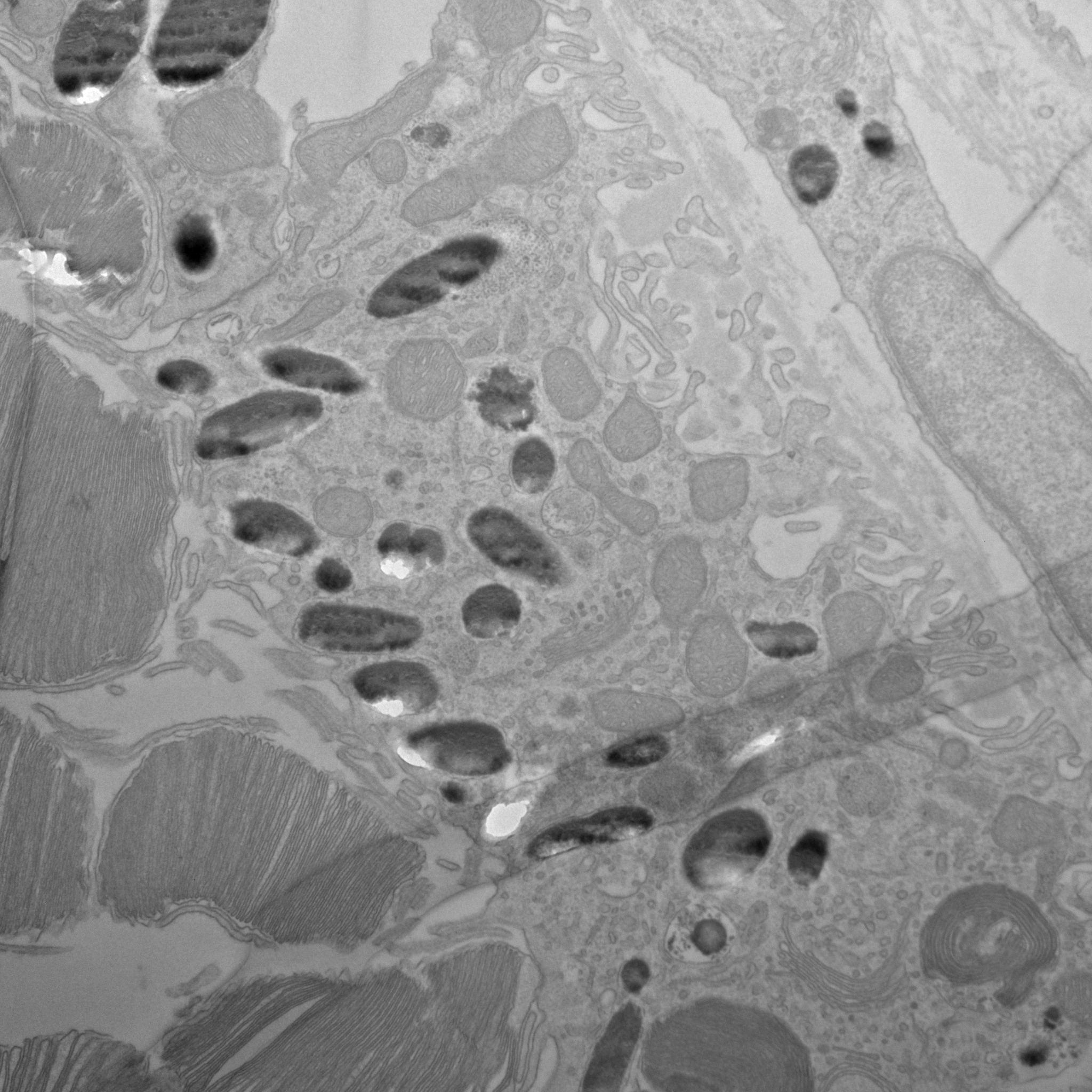

Supplement: Supplementary file 5 — Source Data for Figure 2 [file EMMM-15-e17033-s008.zip › Figure 2/2C/WT 3.tif]

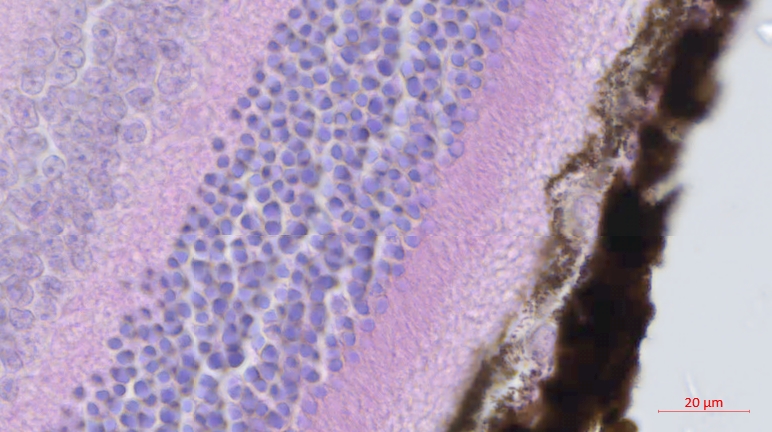

Supplement: Supplementary file 6 — Source Data for Figure 3 [file EMMM-15-e17033-s004.zip › Figure 3/3C/AAV-GFP.jpg]

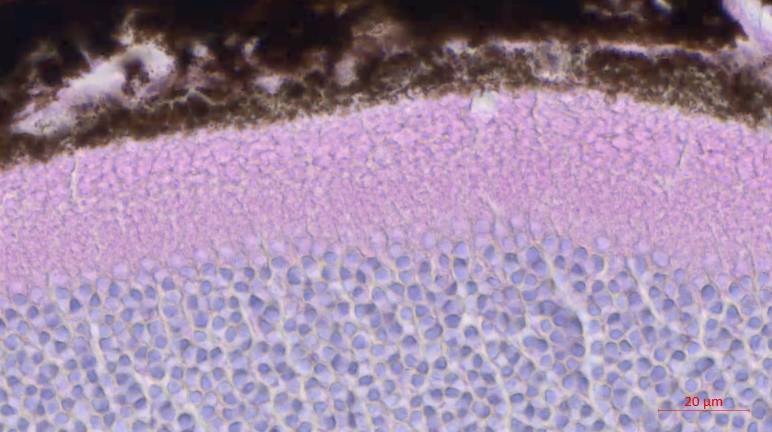

Supplement: Supplementary file 6 — Source Data for Figure 3 [file EMMM-15-e17033-s004.zip › Figure 3/3C/AAV-OAT.jpg]

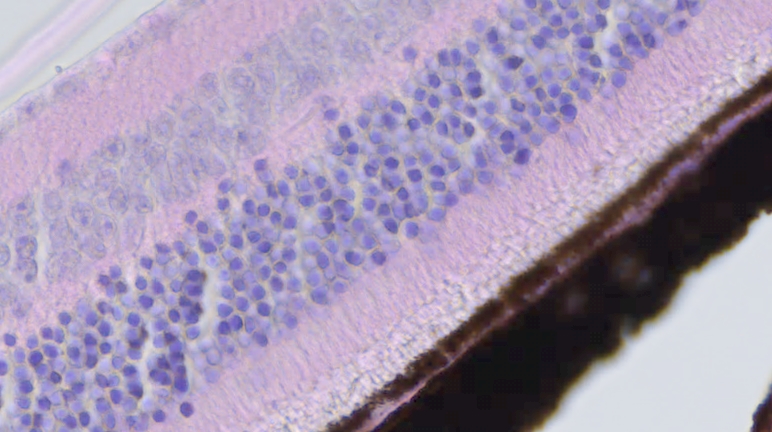

Supplement: Supplementary file 6 — Source Data for Figure 3 [file EMMM-15-e17033-s004.zip › Figure 3/3C/WT.jpg]
