# Supplementary material for: Liver‐directed gene therapy for ornithine aminotransferase deficiency
Source: EMBO Mol Med. 2023 Jan 17;15(4):e17033. doi: 10.15252/emmm.202217033 (PMC10086579; doi:10.15252/emmm.202217033)
Supplement: Supplementary file 9 [file EMMM-15-e17033-s002.pdf]

EMBO Molecular Medicine

Liver-directed gene therapy for ornithine aminotransferase deficiency.

Iolanda Boffa, Elena Polishchuk, Lucia De Stefano, Fabio Dell'Aquila, Edorado Nusco, Elena Marrocco, Matteo Audano, Silvia Pedretti, Marianna Caterino, Ilaria Bellezza, Margherita Ruoppolo, Nico Mitro, Barbara Cellini, Alberto Auricchio, and Nicola Brunetti-Pierri
DOI: 10.15252/emmm.202217033

Corresponding author: Nicola Brunetti-Pierri (brunetti@tigem.it)

Review Timeline:

Submission Date:	14th Oct 22
Editorial Decision:	15th Nov 22
Revision Received:	26th Nov 22
Editorial Decision:	12th Dec 22
Revision Received:	16th Dec 22
Accepted:	19th Dec 22

Editor: Zeljko Durdevic

Transaction Report:

(Note: With the exception of the correction of typographical or spelling errors that could be a source of ambiguity, letters and reports are not edited. Depending on transfer agreements, referee reports obtained elsewhere may or may not be included in this compilation. Referee reports are anonymous unless the Referee chooses to sign their reports.)

15th Nov 2022

Dear Prof. Brunetti-Pierri,

Thank you for the submission of your manuscript to EMBO Molecular Medicine. We have now received feedback from the three reviewers who agreed to evaluate your manuscript. As you will see from the reports, all three referees support publication of the study raising some concerns that should be addressed in a major revision. Data on transduction efficacy and OAT activity in the liver as suggested by the referee #2 should be provided, while the concerns about the effects on urea cycle (referee #1) and cancer risk (referee #2) should be discussed. Safety data are of course welcome but not required.

Further consideration of a revision that addresses reviewers' concerns in full will entail a second round of review. EMBO Molecular Medicine encourages a single round of revision only and therefore, acceptance or rejection of the manuscript will depend on the completeness of your responses included in the next, final version of the manuscript. For this reason, and to save you from any frustrations in the end, I would strongly advise against returning an incomplete revision.

We would welcome the submission of a revised version within three months for further consideration. Please let us know if you require longer to complete the revision.

Please usse this link to login to the manuscript system and submit your revision: https://embomolmed.msubmit.net/cgi-bin/main.plex

I look forward to receiving your revised manuscript.

Yours sincerely,

Zeljko Durdevic

Zeljko Durdevic
Editor
EMBO Molecular Medicine

***** Reviewer's comments *****

Referee #1 (Comments on Novelty/Model System for Author):

This is the first time that it was shown that restoration of hepatic gene expression of OAT can correct the retinal defects of gyrate atrophy. This blindness in this disease is essentially incurable today and the paper provides the rationale for hepatic gene therapy for the disorder. This is a big deal.

Referee #1 (Remarks for Author):

The manuscript by Boffa et al reports partial correction of gyrate atrophy of choroid and retina in a murine model of the disease. They use liver directed gene therapy with an AAV8 vector expressing liver-specific OAT, the enzyme deficient in this disease. Excitingly, this study effectively demonstrates that even partial systemic correction of blood ornithine levels effectively alleviates the vision phenotype of OAT deficiency. This is very promising proof-of-concept data for a disease with few treatment options. Overall, the quality of the data is good. The figures are well done and manuscript is well written.

Specific suggestions for improving the manuscript are given in the following:

Major:

1. OAT is normally tightly specific for zone 3 hepatocytes. The expression of OAT in cells that also express OTC (zone 1 and 2 of the hepatic lobule) could cause a futile cycle and potentially disrupt the urea cycle, causing hyperammonemia. For this reason, the blood levels of all amino acids in blood in treated animals at the 11 month time point should be shown. The blood amino acid data should be in a main figure, not supplementary information.
2. The potential concern of a futile urea cycle from overlap of OAT and OTC expression should be addressed in the discussion. The likely importance of zone 3 restricted expression should be discussed as well.

Minor:

3. Doses for two viruses that are directly compared are given in gc/kg and vp/kg, respectively (page 4-5). Please clarify which is correct.
4. Figure 1a: Add a line indicating physiologically normal ornithine level.
5. Add explanation for those who are not vision specialists to understand how changes in A/B waves correspond to functional changes in vision, and perhaps discussion of how a similar change would impact quality of life for patients
6. Check the figures and legends throughout. Figures 1e-g seem to be out of order and don't correctly correspond to figure legend and in-text references. Figure EV3 seems to have a similar problem. Figure 3c is labeled 3d.
7. The overlap of OAT expression and. GFP shown in EV2 looks too perfect. To me this looks like bleed-through of the channels used for imaging. While it is known that AAV 8 is enriched in pericentral hepatocytes, perfect overlap makes no sense. Please check.

Referee #2 (Remarks for Author):

In this study, the authors demonstrated that AAV8 vector-mediated gene transfer into the hepatocytes ameliorated retinal degeneration in two mice models of ornithine aminotransferase (OAT) deficiency. Since OAT deficiency mainly affects the retina in association with increased concentration of plasma ornithine, it seems a straightforward approach to restoring the OAT activity in the liver where the enzyme functions predominantly in physiologic conditions.

1. What about the transduction efficiency of the liver?
What percent of the hepatocytes in each mouse were transduced?
2. How were the OAT activities in the liver?
OAT expression is regulated by 17b-estradiol and testosterone in physiological conditions. Were there any differences in the OAT activity between male and female mice?
3. OAT is known to be involved in mitotic cell division and is indicated to play a critical role in the progression of hepatocellular carcinoma. In this point of view, it is preferable to demonstrate some safety data that the overexpression of OAT in the hepatocytes did not increase the tumor risk.

Ref. Montioli R, et al. Deficit of human ornithine aminotransferase in gyrate atrophy: Molecular, cellular, and clinical aspects. Biochim Biophys Acta Proteins Proteom. 2021;1869(1):140555.

Minor points
1. page 6, line 13; AVV-GFP → AAV-GFP
2. page 9, AAV2/8.TBG.GFP; spell out TBG promoter as thyroxine-binding globulin promoter

Referee #3 (Comments on Novelty/Model System for Author):

First proof of concept study for liver directed AAV based gene therapy study for OAT.
Suitable for publication in current format.
The work is of high quality and I only have minor comments for improvements:
1. Introduction:
Line 9 should say: "deficiency in synthesis OF ornithine"
2. Page 5 of the PDF refers to EV3h: I cannot find the EV3h as EV figure finishes with g.
3. Figure one is mixed up. When it is referred to in the text on page 6. 1e is the western blot. When it refers to 1f-g. it should be e-f.
4. Discussion
should say "The only available treatment is a stringent diet with poor patient adherence".

Referee #3 (Remarks for Author):

The work is of high quality and I only have minor comments for improvements:
1. Introduction:
Line 9 should say: "deficiency in synthesis OF ornithine"
2. Page 5 of the PDF refers to EV3h: I cannot find the EV3h as EV figure finishes with g.
3. Figure one is mixed up. When it is referred to in the text on page 6. 1e is the western blot. When it refers to 1f-g. it should be e-f.
4. Discussion
should say "The only available treatment is a stringent diet with poor patient adherence".

Point-by-point response to the Editor and Reviewers' comments

As you will see from the reports, all three referees support publication of the study raising some concerns that should be addressed in a major revision. Data on transduction efficacy and OAT activity in the liver as suggested by the referee #2 should be provided, while the concerns about the effects on urea cycle (referee #1) and cancer risk (referee #2) should be discussed. Safety data are of course welcome but not required.

Authors' response: We thank the Editor and the reviewers for their positive feedback. To address the points raised by the reviewers, we evaluated transduction of livers by AAV vectors and we included this new data as fig. 1g. Please note that the activity of OAT was previously performed (data shown in fig. 1f). We also discussed the potential detrimental effects of OAT gene transfer on the urea cycle. Finally, we briefly mentioned the cancer risk but, as stated in the discussion this would be an issue in case of OAT overexpression.

Referee #1

This is the first time that it was shown that restoration of hepatic gene expression of OAT can correct the retinal defects of gyrate atrophy. This blindness in this disease is essentially incurable today and the paper provides the rationale for hepatic gene therapy for the disorder. This is a big deal.

Authors' response: We thank the reviewers for the positive feedback.

The manuscript by Boffa et al reports partial correction of gyrate atrophy of choroid and retina in a murine model of the disease. They use liver directed gene therapy with an AAV8 vector expressing liver-specific OAT, the enzyme deficient in this disease. Excitingly, this study effectively demonstrates that even partial systemic correction of blood ornithine levels effectively alleviates the vision phenotype of OAT deficiency. This is very promising proof-of-concept data for a disease with few treatment options. Overall, the quality of the data is good. The figures are well done and manuscript is well written.

Authors' response: We thank the reviewers for the positive feedback.

1. OAT is normally tightly specific for zone 3 hepatocytes. The expression of OAT in cells that also express OTC (zone 1 and 2 of the hepatic lobule) could cause a futile cycle and potentially disrupt the urea cycle, causing hyperammonemia. For this reason, the blood levels of all amino acids in blood in treated animals at the 11 month time point should be shown. The blood amino acid data should be in a main figure, not supplementary information.

Authors' response: We included the data on plasma amino acid concentrations as table 1 in the main manuscript whereas the disease-relevant amino acids remain in the figure 1.

2. The potential concern of a futile urea cycle from overlap of OAT and OTC expression should be addressed in the discussion. The likely importance of zone 3 restricted expression should be discussed as well.

Authors' response: this concern about futile urea cycle has been included in the discussion.

Minor:

3. Doses for two viruses that are directly compared are given in gc/kg and vp/kg, respectively (page 4-5). Please clarify which is correct.
Authors' response: We thank the reviewer for noting this inconsistency that we fixed.

4. Figure 1a: Add a line indicating physiologically normal ornithine level.
Authors' response: the normal range of plasma ornithine has been included (see revised Figs. 1 and 3, and revised Expanded View Figures).

5. Add explanation for those who are not vision specialists to understand how changes in A/B waves correspond to functional changes in vision, and perhaps discussion of how a similar change would impact quality of life for patients.
Authors' response: this explanation has been included.

6. Check the figures and legends throughout. Figures 1e-g seem to be out of order and don't correctly correspond to figure legend and in-text references. Figure EV3 seems to have a similar problem. Figure 3c is labeled 3d.
Authors' response: We apologize for the mistake that we have corrected. We also carefully checked all figures and EV figures and legends.

7. The overlap of OAT expression and. GFP shown in EV2 looks too perfect. To me this looks like bleed-through of the channels used for imaging. While it is known that AAV 8 is enriched in pericentral hepatocytes, perfect overlap makes no sense. Please check.
Authors' response: We changed the images with new images showing still a great overlap of the fluorescence signals but also showing cells that are positive for just one of the two signals.

Referee #2
1. What about the transduction efficiency of the liver? What percent of the hepatocytes in each mouse were transduced?
Authors' response: we performed OAT immunohistochemistry to show the hepatic transduction following AAV-OAT delivery.

2. How were the OAT activities in the liver? OAT expression is regulated by 17b-estradiol and testosterone in physiological conditions. Were there any differences in the OAT activity between male and female mice?
Authors' response: OAT activity was included in fig. 1f. The OAT gene delivered by the AAV vector is under the control of the TBG promoter and not under the control of its endogenous promoter. Therefore, the physiologic regulation by factors binding to the OAT promoter is not active in the context of this gene therapy study. We did not observe significant differences in OAT activity of wild-type male and female mice, as shown in the figure on the right.

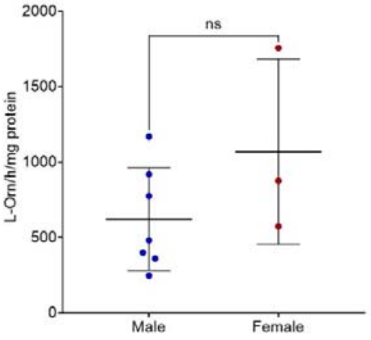

Figure. OAT activity in livers of male and female wild-type C57B6 mice.

3. OAT is known to be involved in mitotic cell division and is indicated to play a critical role in the progression of hepatocellular carcinoma. In this point of view, it is preferable to demonstrate some safety data that the overexpression of OAT in the hepatocytes did not increase the tumor risk.

Authors' response: In our experiments OAT was not overexpressed and indeed the expression achieved is below physiologic levels. The theoretical risk of hepatocellular carcinoma might be considered when more than 100% of normal activity is obtained. We have included this point in the discussion.

Minor points
1. page 6, line 13; AVV-GFP → AAV-GFP
2. page 9, AAV2/8.TBG.GFP; spell out TBG promoter as thyroxine-binding globulin promoter
Authors' response: We corrected the typo on page 6 and the TBG promoter has been spelled out the time it appears in the text.

Referee #3
First proof of concept study for liver directed AAV based gene therapy study for OAT. Suitable for publication in current format. The work is of high quality and I only have minor comments for improvements:

1. Introduction: Line 9 should say: "deficiency in synthesis OF ornithine".
Authors' response: Corrected.

2. Page 5 of the PDF refers to EV3h: I cannot find the EV3h as EV figure finishes with g.
Authors' response: Corrected.

3. Figure one is mixed up. When it is referred to in the text on page 6. 1e is the western blot. When it refers to 1f-g. it should be e-f.
Authors' response: We apologize for the oversight, and we have fixed the mistake.

4. Discussion should say "The only available treatment is a stringent diet with poor patient adherence".
Authors' response: This has been corrected.

12th Dec 2022

Dear Prof. Brunetti-Pierri,

Thank you for the submission of your revised manuscript to EMBO Molecular Medicine. I am pleased to inform you that we will be able to accept your manuscript pending the following final amendments:

1) Please address the referees' minor suggestions.
2) Authors: Margherita Ruoppolo is not entered in our submission system. Please make sure that information for all authors are entered in our submission system.
3) Figures: Please upload high-resolution main Figure files. Please check "Author Guidelines" for more information:
https://www.embopress.org/page/journal/17574684/authorguide#figureformat
4) EV Figures: Please upload individual, high-resolution EV Figure files and place their legends in the main manuscript file after the main figure legends.
5) Tables: Please upload individual, editable files for both Table 1 Table EV1 with their legend added to the table.
6) In the main manuscript file, please do the following:
- Correct/answer the track changes suggested by our data editors by working from the attached document.
- Add up to 5 keywords.
- In M&M, the statistical paragraph should reflect all information that you have filled in the Authors Checklist, especially regarding randomization, blinding, replication.
- Please rename "Competing Interest" to "Disclosure Statement & Competing Interests". We updated our journal's competing interests policy in January 2022 and request authors to consider both actual and perceived competing interests. Please review the policy https://www.embopress.org/competing-interests and update your competing interests if necessary.
- Author contributions: Please remove it from the manuscript and specify author contributions in our submission system. CRediT has replaced the traditional author contributions section because it offers a systematic machine-readable author contributions format that allows for more effective research assessment. You are encouraged to use the free text boxes beneath each contributing author's name to add specific details on the author's contribution. More information is available in our guide to authors:
https://www.embopress.org/page/journal/17574684/authorguide#authorshipguidelines
- Add data availability statement. If no data are deposited in public repositories, please add the sentence: "This study includes no data deposited in external repositories".
Please check "Author Guidelines" for more information.
https://www.embopress.org/page/journal/17574684/authorguide#availabilityofpublishedmaterial
7) Supplemental M&M: Please add all M&M to the main manuscript file.
8) Funding: Please make sure that information about all sources of funding are complete in both our submission system and in the manuscript.
9) The Paper Explained: Please provide a summary of the study structured as followed: PROBLEM - the medical issue you are addressing, RESULTS - the results obtained, and IMPACT - their clinical impact. Please refer to any of our published primary research articles for an example. Please check "Author Guidelines" for more information.
https://www.embopress.org/page/journal/17574684/authorguide#researcharticleguide
10) Synopsis: Every published paper now includes a 'Synopsis' to further enhance discoverability. Synopses are displayed on the journal webpage and are freely accessible to all readers. They include separate synopsis image and synopsis text.
- Synopsis image: Please provide a striking image or visual abstract as a high-resolution jpeg file 550 px-wide x (250-400)-px high to illustrate your article.
- Synopsis text: Please provide a short standfirst (maximum of 300 characters, including space) as well as 2-5 one sentence bullet points that summarise the paper as a .doc file. Please write the bullet points to summarise the key NEW findings. They should be designed to be complementary to the abstract - i.e. not repeat the same text. We encourage inclusion of key acronyms and quantitative information (maximum of 30 words / bullet point). Please use the passive voice.
- Please check your synopsis text and image before submission with your revised manuscript. Please be aware that in the proof stage minor corrections only are allowed (e.g., typos).
11) For more information: This space should be used to list relevant web links for further consultation by our readers. Could you identify some relevant ones and provide such information as well? Some examples are patient associations, relevant databases, OMIM/proteins/genes links, author's websites, etc...
12) Source data: Please upload source data as clearly labelled one (zipped) file per figure.
13) As part of the EMBO Publications transparent editorial process initiative (see our Editorial at http://embomolmed.embopress.org/content/2/9/329), EMBO Molecular Medicine will publish online a Review Process File (RPF) to accompany accepted manuscripts. This file will be published in conjunction with your paper and will include the anonymous referee reports, your point-by-point response and all pertinent correspondence relating to the manuscript. Let us know whether you agree with the publication of the RPF and as here, if you want to remove or not any figures from it prior to publication. Please note that the Authors checklist will be published at the end of the RPF.
14) Please provide a point-by-point letter INCLUDING my comments as well as the reviewer's reports and your detailed responses (as Word file).

I look forward to reading a new revised version of your manuscript as soon as possible.

Yours sincerely,

Zeljko Durdevic

Zeljko Durdevic
Editor
EMBO Molecular Medicine

***** Reviewer's comments *****

Referee #1 (Comments on Novelty/Model System for Author):

Two separate mouse models of gyrate atrophy were used and they both establish a clear link between systemically elevated ornithine levels and retinal degeneration. This is the best preclinical model of the human disease available.

Referee #1 (Remarks for Author):

The authors have adequately addressed my concerns. I also think that they have adequately addressed the other reviewer's questions as well. This publication will bring hope to patients with gyrate atrophy, who really have had an incurable condition until now.

Minor suggestions:

- In the discussion, use the term "complete normalization" of ornithine levels instead of just "normalization". I think that emphasizes your point better.
- "steeled" (in the discussion of urea cycle depletion) should be "stolen" or "removed from".

Referee #2 (Remarks for Author):

The authors added immunohistochemistry of the liver.
It is preferable to provide quantitative data such as the numbers of the transduced cells.

The authors addressed the minor editorial issues.

We are pleased to inform you that your manuscript is accepted for publication and is now being sent to our publisher to be included in the next available issue of EMBO Molecular Medicine.

EMBO Press Author Checklist

Corresponding Author Name: Ana Martin-Villalba, Anna Marciniak-Czochra
Journal Submitted to: EMBO Molecular Medicine
Manuscript Number: EMM-2022-16434

USEFUL LINKS FOR COMPLETING THIS FORM
[The EMBO Journal - Author Guidelines]
[EMBO Reports - Author Guidelines]
[Molecular Systems Biology - Author Guidelines]
[EMBO Molecular Medicine - Author Guidelines]

Reporting Checklist for Life Science Articles (updated January 2022)

This checklist is adapted from Materials Design Analysis Reporting (MDAR) Checklist for Authors. MDAR establishes a minimum set of requirements in transparent reporting in the life sciences (see Statement of Task: 10.31222/osf.io/9sm4x). Please follow the journal's guidelines in preparing your manuscript.

Please note that a copy of this checklist will be published alongside your article.

Abridged guidelines for figures

1. Data

The data shown in figures should satisfy the following conditions:

- the data were obtained and processed according to the field's best practice and are presented to reflect the results of the experiments in an accurate and unbiased manner.
- ideally, figure panels should include only measurements that are directly comparable to each other and obtained with the same assay.
- plots include clearly labeled error bars for independent experiments and sample sizes. Unless justified, error bars should not be shown for technical replicates.
- if n<5, the individual data points from each experiment should be plotted. Any statistical test employed should be justified.
- Source Data should be included to report the data underlying figures according to the guidelines set out in the authorship guidelines on Data Presentation.

2. Captions

Each figure caption should contain the following information, for each panel where they are relevant:

- a specification of the experimental system investigated (eg cell line, species name).
- the assay(s) and method(s) used to carry out the reported observations and measurements.
- an explicit mention of the biological and chemical entity(ies) that are being measured.
- an explicit mention of the biological and chemical entity(ies) that are altered/varied/perturbed in a controlled manner.
- the exact sample size (n) for each experimental group/condition, given as a number, not a range;
- a description of the sample collection allowing the reader to understand whether the samples represent technical or biological replicates (including how many animals, litters, cultures, etc.).
- a statement of how many times the experiment shown was independently replicated in the laboratory.
- definitions of statistical methods and measures:
  - common tests, such as t-test (please specify whether paired vs. unpaired), simple χ2 tests, Wilcoxon and Mann-Whitney tests, can be unambiguously identified by name only, but more complex techniques should be described in the methods section;
  - are tests one-sided or two-sided?
  - are there adjustments for multiple comparisons?
  - exact statistical test results, e.g., P values = x but not P values < x;
  - definition of 'center values' as median or average;
  - definition of error bars as s.d. or s.e.m.

Please complete ALL of the questions below.
Select "Not Applicable" only when the requested information is not relevant for your study.

Materials

Newly Created Materials	Information included in the manuscript?	In which section is the information available? (Reagents and Tools Table, Materials and Methods, Figures, Data Availability Section)
New materials and reagents need to be available; do any restrictions apply?	Yes	Data availability

Antibodies	Information included in the manuscript?	In which section is the information available? (Reagents and Tools Table, Materials and Methods, Figures, Data Availability Section)
For **antibodies** provide the following information:<br>- Commercial antibodies: RRID (if possible) or supplier name, catalogue number and or/clone number<br>- Non-commercial: RRID or citation	Yes	Materials and Methods

DNA and RNA sequences	Information included in the manuscript?	In which section is the information available? (Reagents and Tools Table, Materials and Methods, Figures, Data Availability Section)
**Short novel DNA or RNA including primers, probes**: provide the sequences.	Yes	Materials and Methods, Expanded View tables

Cell materials	Information included in the manuscript?	In which section is the information available? (Reagents and Tools Table, Materials and Methods, Figures, Data Availability Section)
**Cell lines:** Provide species information, strain. Provide accession number in repository **OR** supplier name, catalog number, clone number, and/**OR** RRID.	Not Applicable	
**Primary cultures:** Provide species, strain, sex of origin, genetic modification status.	Yes	Materials and Methods
Report if the cell lines were recently **authenticated** (e.g., by STR profiling) and tested for mycoplasma contamination.	Not Applicable	

Experimental animals	Information included in the manuscript?	In which section is the information available? (Reagents and Tools Table, Materials and Methods, Figures, Data Availability Section)
**Laboratory animals or Model organisms:** Provide species, strain, sex, age, genetic modification status. Provide accession number in repository **OR** supplier name, catalog number, clone number, **OR** RRID.	Yes	Materials and Methods
**Animal observed in or captured from the field:** Provide species, sex, and age where possible.	Not Applicable	
Please detail **housing and husbandry conditions**.	Yes	Materials and Methods

Plants and microbes	Information included in the manuscript?	In which section is the information available? (Reagents and Tools Table, Materials and Methods, Figures, Data Availability Section)
**Plants:** provide species and strain, ecotype and cultivar where relevant, unique accession number if available, and source (including location for collected wild specimens).	Not Applicable	
**Microbes:** provide species and strain, unique accession number if available, and source.	Not Applicable	

Human research participants	Information included in the manuscript?	In which section is the information available? (Reagents and Tools Table, Materials and Methods, Figures, Data Availability Section)
If collected and within the bounds of privacy constraints report on age, sex and gender or ethnicity for all study participants.	Not Applicable	

Core facilities	Information included in the manuscript?	In which section is the information available? (Reagents and Tools Table, Materials and Methods, Figures, Data Availability Section)
If your work benefited from core facilities, was their service mentioned in the acknowledgments section?	Yes	Acknowledgments

Design

Study protocol	Information included in the manuscript?	In which section is the information available? (Reagents and Tools Table, Materials and Methods, Figures, Data Availability Section)
If study protocol has been **pre-registered, provide DOI in the manuscript**. For clinical trials, provide the trial registration number **OR** cite DOI.	Not Applicable	
Report the **clinical trial registration number** (at ClinicalTrials.gov or equivalent), where applicable.	Not Applicable	

Laboratory protocol	Information included in the manuscript?	In which section is the information available? (Reagents and Tools Table, Materials and Methods, Figures, Data Availability Section)
Provide DOI OR other citation details if **external detailed step-by-step protocols** are available.	Not Applicable	

Experimental study design and statistics	Information included in the manuscript?	In which section is the information available? (Reagents and Tools Table, Materials and Methods, Figures, Data Availability Section)
Include a statement about **sample size** estimate even if no statistical methods were used.	Not Applicable	
Were any steps taken to minimize the effects of subjective bias when allocating animals/samples to treatment (e.g. **randomization procedure**)? If yes, have they been described?	Not Applicable	
Include a statement about **blinding** even if no blinding was done.	Not Applicable	
Describe **inclusion/exclusion criteria** if samples or animals were excluded from the analysis. Were the criteria pre-established? <br>If sample or data points were omitted from analysis, report if this was due to attrition or intentional exclusion and provide justification.	Yes	Materials and Methods, Figure captions
For every figure, are **statistical tests** justified as appropriate? Do the data meet the assumptions of the tests (e.g., normal distribution)? Describe any methods used to assess it. Is there an estimate of variation within each group of data? Is the variance similar between the groups that are being statistically compared?	Yes	Materials and Methods, Figure captions

Sample definition and in-laboratory replication	Information included in the manuscript?	In which section is the information available? (Reagents and Tools Table, Materials and Methods, Figures, Data Availability Section)
In the figure legends: state number of times the experiment was **replicated** in laboratory.	Yes	Figure captions
In the figure legends: define whether data describe **technical or biological replicates**.	Yes	Figure captions

Ethics

Ethics	Information included in the manuscript?	In which section is the information available? (Reagents and Tools Table, Materials and Methods, Figures, Data Availability Section)
Studies involving **human participants**: State details of **authority granting ethics approval** (IRB or equivalent committee(s), provide reference number for approval.	Not Applicable	
Studies involving **human participants**: Include a statement confirming that **informed consent** was obtained from all subjects and that the experiments conformed to the principles set out in the WMA Declaration of Helsinki and the Department of Health and Human Services Belmont Report.	Not Applicable	
Studies involving **human participants:** For publication of **patient photos**, include a statement confirming that consent to publish was obtained.	Not Applicable	
Studies involving experimental **animals**: State details of **authority granting ethics approval** (IRB or equivalent committee(s), provide reference number for approval. Include a statement of compliance with ethical regulations.	Yes	Materials and Methods
Studies involving **specimen and field samples:** State if relevant **permits** obtained, provide details of authority approving study; if none were required, explain why.	Not Applicable	

Dual Use Research of Concern (DURC)	Information included in the manuscript?	In which section is the information available? (Reagents and Tools Table, Materials and Methods, Figures, Data Availability Section)
Could your study fall under dual use research restrictions? Please check biosecurity documents and list of **select agents and toxins** (CDC): https://www.selectagents.gov/sat/list.htm	Not Applicable	
If you used a select agent, is the security level of the lab appropriate and reported in the manuscript?	Not Applicable	
If a study is subject to dual use research of concern regulations, is the name of the **authority granting approval and reference number** for the regulatory approval provided in the manuscript?	Not Applicable	

Reporting

The MDAR framework recommends adoption of discipline-specific guidelines, established and endorsed through community initiatives. Journals have their own policy about requiring specific guidelines and recommendations to complement MDAR.

Adherence to community standards	Information included in the manuscript?	In which section is the information available? (Reagents and Tools Table, Materials and Methods, Figures, Data Availability Section)
State if relevant guidelines or checklists (e.g., **ICMJE, MIBBI, ARRIVE, PRISMA**) have been followed or provided.	Not Applicable	
For **tumor marker prognostic studies,** we recommend that you follow the **REMARK** reporting guidelines (see link list at top right). See author guidelines, under 'Reporting Guidelines'. Please confirm you have followed these guidelines.	Not Applicable	
For **phase II and III randomized controlled trials**, please refer to the **CONSORT** flow diagram (see link list at top right) and submit the CONSORT checklist (see link list at top right) with your submission. See author guidelines, under 'Reporting Guidelines'. Please confirm you have submitted this list.	Not Applicable	

Data Availability

Data availability	Information included in the manuscript?	In which section is the information available? (Reagents and Tools Table, Materials and Methods, Figures, Data Availability Section)
Have **primary datasets** been deposited according to the journal's guidelines (see 'Data Deposition' section) and the respective accession numbers provided in the Data Availability Section?	Yes	Materials and Methods; Data Availability Section
Were **human clinical and genomic datasets** deposited in a public access-controlled repository in accordance to ethical obligations to the patients and to the applicable consent agreement?	Not Applicable	
Are **computational models** that are central and integral to a study available without restrictions in a machine-readable form? Were the relevant accession numbers or links provided?	Yes	Materials and Methods; Data Availability Section
If publicly available data were reused, provide the respective **data citations in the reference list**.	Not Applicable	
